# Mapping regional implementation of ‘Making Every Contact Count’: mixed-methods evaluation of implementation stage, strategies, barriers and facilitators of implementation

**DOI:** 10.1136/bmjopen-2024-084208

**Published:** 2024-07-22

**Authors:** Angela M Rodrigues, Bethany Nichol, Rob Wilson, Caroline Charlton, Beckie Gibson, Tracy Finch, Catherine Haighton, Gregory Maniatopoulos, Emma Giles, Deborah Harrison, Denise Orange, Craig Robson, Jill Harland

**Affiliations:** 1 Department of Psychology, Northumbria University, Newcastle upon Tyne, UK; 2 Department of Social Work, Education and Community Wellbeing, Northumbria University Newcastle, Newcastle Upon Tyne, UK; 3 Newcastle Business School, Northumbria University, Newcastle upon Tyne, UK; 4 Department of Nursing, Midwifery and Health, Northumbria University, Newcastle upon Tyne, UK; 5 University of Leicester School of Business, University of Leicester, Leicester, UK; 6 School of Health and Life Sciences, Teesside University, Middlesbrough, UK; 7 Newcastle University Business School, Newcastle University, Newcastle upon Tyne, UK; 8 Office for Health Improvement & Disparities, Newcastle upon Tyne, UK; 9 North Tyneside General Hospital, Northumbria Healthcare NHS Foundation Trust, North Shields, UK

**Keywords:** health services, qualitative research, public health, implementation science, primary prevention

## Abstract

**Abstract:**

**Background:**

The Making Every Contact Count (MECC) programme provides training and materials to support public-facing workers to encourage health-promoting behaviour change by using the day-to-day interactions between organisations and individuals. This project aimed to analyse MECC implementation through a comparative analysis of implementation stage, strategies used for implementation and enablers/barriers of the implementation process within a region in England—the North East and North Cumbria (NENC).

**Methods:**

A mixed-methods process evaluation was conducted applying normalisation process theory and theoretical domains framework. MECC programme documents were reviewed and mapped against specific criteria (eg, implementation strategies). An online mapping survey was conducted to establish current implementation/delivery of MECC within NENC settings (eg, local government, healthcare and voluntary community sector). Qualitative research, using individual interviews and group discussions, was conducted to establish further understanding of MECC implementation.

**Results:**

Our findings were informed by reviewing documents (n=5), surveying participants (n=34), interviews (n=18) and group discussions (n=48). Overall, the implementation of MECC within the region was at an early stage, with training mostly delivered between, rather than within, organisations. Qualitative findings highlighted factors that influence stakeholders to implement MECC (eg, organisational goals that were facilitated by MECC implementation, including the prevention agenda), supported resources that facilitate the implementation of MECC (eg, logic models) and enabling factors that promote MECC sustainability across the region (eg, buy-in from leadership and management).

**Conclusions:**

The NENC MECC programme is built around regional leadership that supports the implementation process. This process evaluation identified key influences of MECC implementation across the region. We discuss evidence-based recommendation for policy and practice that can be taken forward to develop targeted strategies to support future MECC implementation. For example, a co-ordinated infrastructure and strategy is needed to combat delivery and implementation issues identified.

**STRENGTHS AND LIMITATIONS OF THIS STUDY**

- The Making Every Contact Count (MECC) regional strategy group played a pivotal role acting in an advisory capacity.
- Our findings informed recommendations to strategically optimise MECC implementation.
- Our systematic approach yields evidence facilitating efficient translation to policy and practice.
- Recruitment challenges limited our dataset, which may not capture the regional MECC diversity.

## Introduction

 Several long-term diseases (eg, diabetes, cancer and cardiovascular diseases) are directly associated with known behavioural risk factors.^1^ In the UK, approximately 40% of disability-adjusted life-years lost are attributable to tobacco, poor diet, alcohol or being physically inactive.^2^ Supporting people to make changes such as stopping smoking, improving diet, increasing physical activity, maintaining a healthy weight and reducing alcohol consumption should help to reduce their risk of poor health.^2^ There is evidence to support the effectiveness of brief interventions in encouraging people to adopt healthier behaviours including smoking cessation,^3^ physical activity,^4^ healthy eating,^5^ alcohol consumption^6^ and drug use.^7^

Making Every Contact Count (MECC) is a national, long-term public health strategy with its roots in attempts to improve clinicians’ skills in delivering behavioural change interventions to patients.^8^,^10^ Rolled out by NHS (National Health Service) Trusts and local government in England since 2010, MECC provides training and materials to support public-facing workers to opportunistically encourage people to consider healthy behaviour change during routine health and social care contacts.^10^ MECC is central to the UK Government’s prevention agenda and early intervention strategies in public health.^11^ MECC aims to respond to significant health outcomes and pressure on services created by lifestyle choices as a potentially cost effective way of improving health.^12^ Public Health England^13 14^ and Alcohol Change UK^15^ estimated annual costs to the NHS of ~£12.1 billion for some health-related behaviour (ie, smoking, alcohol use, obesity). Since 2016, the MECC approach has been expanded to include wider determinants of health,^10^ which is known as MECC plus. This may include conversations about wider determinants such as debt management, housing and welfare rights advice. Despite this broader definition of MECC, a potential shortcoming is that MECC remains an individualised lens to behaviour change (eg, debt and food poverty are felt at household/community-level rather than individual) and implemented primarily by single organisations.

Underpinned by behaviour change theory, MECC capitalises on the opportunity within routine health and social interactions for brief (lasting between five and thirty minutes) or very brief (lasting seconds to 5 min) interventions on health or well-being factors to take place. Although conceptualised in different ways in the literature, these ‘teachable moments’ are considered to be opportunities or contexts/events representing an increased desire, willingness or capacity for change in people.^16 17^ An MECC conversation can provide that teachable moment for behaviour change, alongside well-timed strategies to promote change. The ultimate aim of MECC is to give people opportunities and resources to make health-related behaviour change in a wide range of settings within and beyond the NHS.^10 18^ MECC training has been shown to be effective in improving and increasing use of client-centred skills to support behaviour change among health and social care practitioners.^19^

MECC lacks a universal approach, as organisations adopt diverse strategies, potentially contributing to a scarcity of evidence regarding its effectiveness and to disparities in implementation success.^20^ Existing studies explore experience of MECC implementation, including frontline worker perceptions and organisational challenges.^21^,^23^ Qualitative interviews with stakeholders engaged in the delivery of MECC suggest that the take-up is variable across different organisations.^24^ Further qualitative work has highlighted that healthcare professionals in the UK felt limited by their work environment to deliver brief interventions (eg, busy workload, lack of appropriate settings conducive to having discussions about behaviour change).^25^ A recent survey conducted in Ireland^26^ identified potential intervention targets for implementation interventions to enhance MECC delivery, such as addressing healthcare professionals’ intentions and goals, barriers to prioritisation, environmental resources, beliefs about capabilities, negative emotions and skills. An evaluation of a local MECC programme demonstrated the importance of implementation and delivery models in shaping MECC characteristics (eg, programme reach/diversity, opportunities and barriers) and the type and nature of MECC interventions to service users.^18^ Findings also illustrated the role of wider factors (eg, organisational culture, economic/political context) in shaping MECC implementation.^18^ Furthermore, wide differences in reporting/monitoring systems between delivery partners highlighted barriers to large-scale outcome evaluation which require attention during implementation.^18^

Notably, there is a lack of comparative studies that span different sectors, delivery models, geographical locations or demographic groups, or that examine relationships between organisations and other actors. This prevents critical comparison of variation in MECC implementation, including aspects such as programme reach and nature of client contact. The lack of information about MECC delivery at a system level also provides a practical barrier to joined up working by various partners including local government, healthcare and the voluntary and community sector (VCS). Evidence suggests great variation in how MECC training is delivered, resulting in a push for MECC training to be standardised as a way to enhance MECC implementation.^27^ As such, it is relevant to strengthen the evidence base underpinning MECC and staff training by clarifying how variations impact the implementation process.

To maximise the impact of MECC, there is a need to explore the implementation process and identify scope for its optimisation. A broad, system-level understanding that integrates different implementation models and approaches represents a unique and important step towards strengthening the evidence base, understanding good practice and responding to identified priorities. It will support stakeholders and the wider public health community to take an informed, strategic approach to continued MECC implementation. We chose theoretical frameworks that offer complementary insights to understand and explain the implementation of MECC, including social processes (normalisation process theory (NPT)) and determinants of behaviour (theoretical domains framework (TDF)). The TDF, a synthesis framework of factors known to influence behaviour change,^28 29^ is employed to facilitate identification of cognitive, affective, social and environmental barriers and facilitators to MECC implementation. The NPT^30 31^ is a sociological theory of implementation that has been used to explain how implementation takes place, with reference to the collaborative work involved in implementation.^32^ NPT proposes that successful implementation is more likely when participants value the intervention (coherence), commit to engage (cognitive participation), commit staff and resources and work towards change (collective action), and appraise the package as useful (reflexive monitoring).^31^

The aim of the study was to explore MECC implementation and delivery across a large geographical region in the north of England serving a population of 3 million, through a comparative analysis of implementation stage, strategies used for implementation and enablers/barriers of the implementation process.

## Methods

### Study design

**Figure 1:**
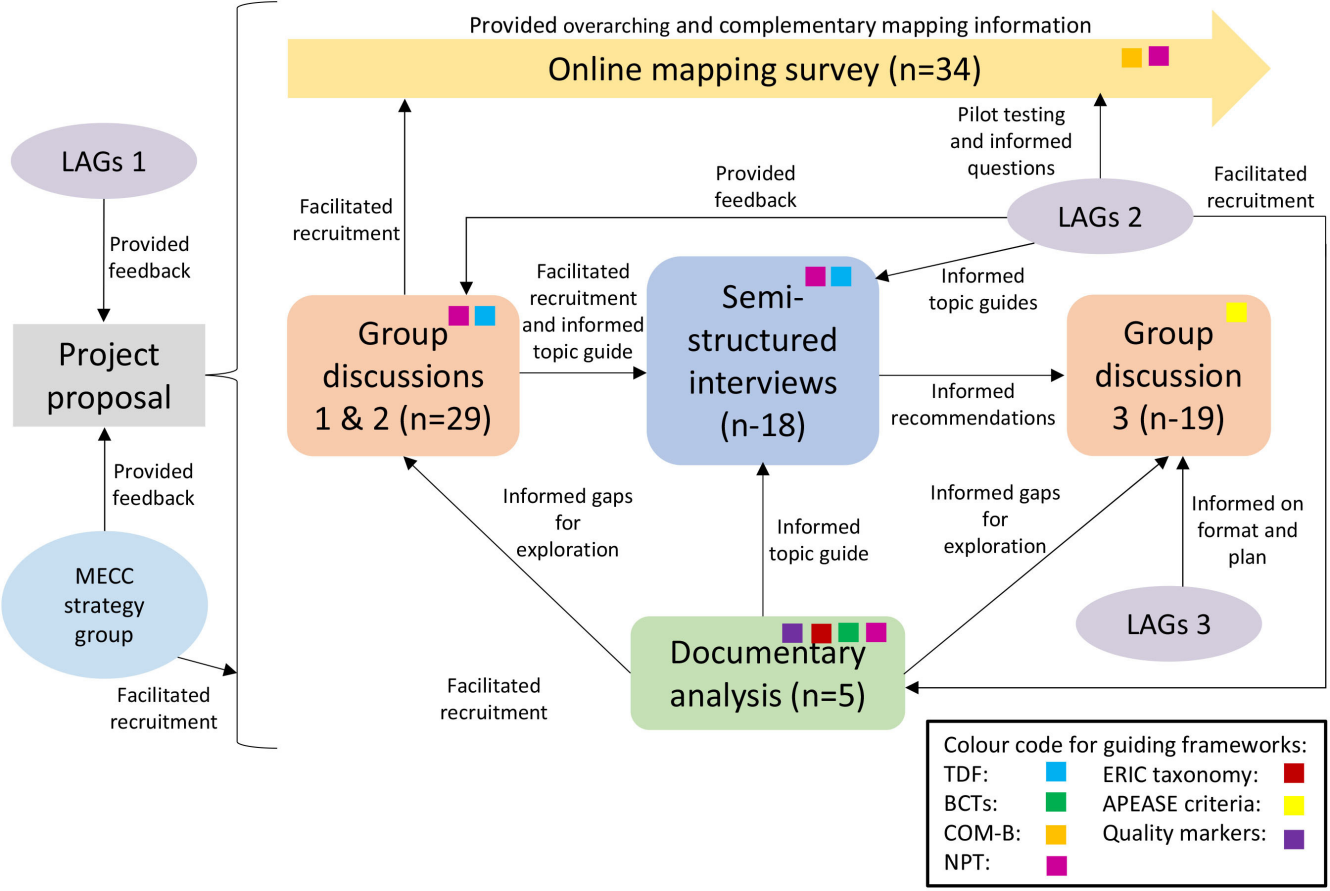
Visual map of study design. For the first LAG meetings, the two groups met separately and for the subsequent two meetings the groups met together. APEASE, affordable, practical, effective, acceptable, safe and equitable; BCTs, behaviour change techniques; COM-B, Capability, Opportunity, Motivation and Behaviour; ERIC, Expert Recommendations for Implementing Change strategies; LAGs, lay advisory groups; MECC, Making Every Contact Count; NPT, normalisation process theory; TDF, theoretical domains framework.

**Figure 2:**
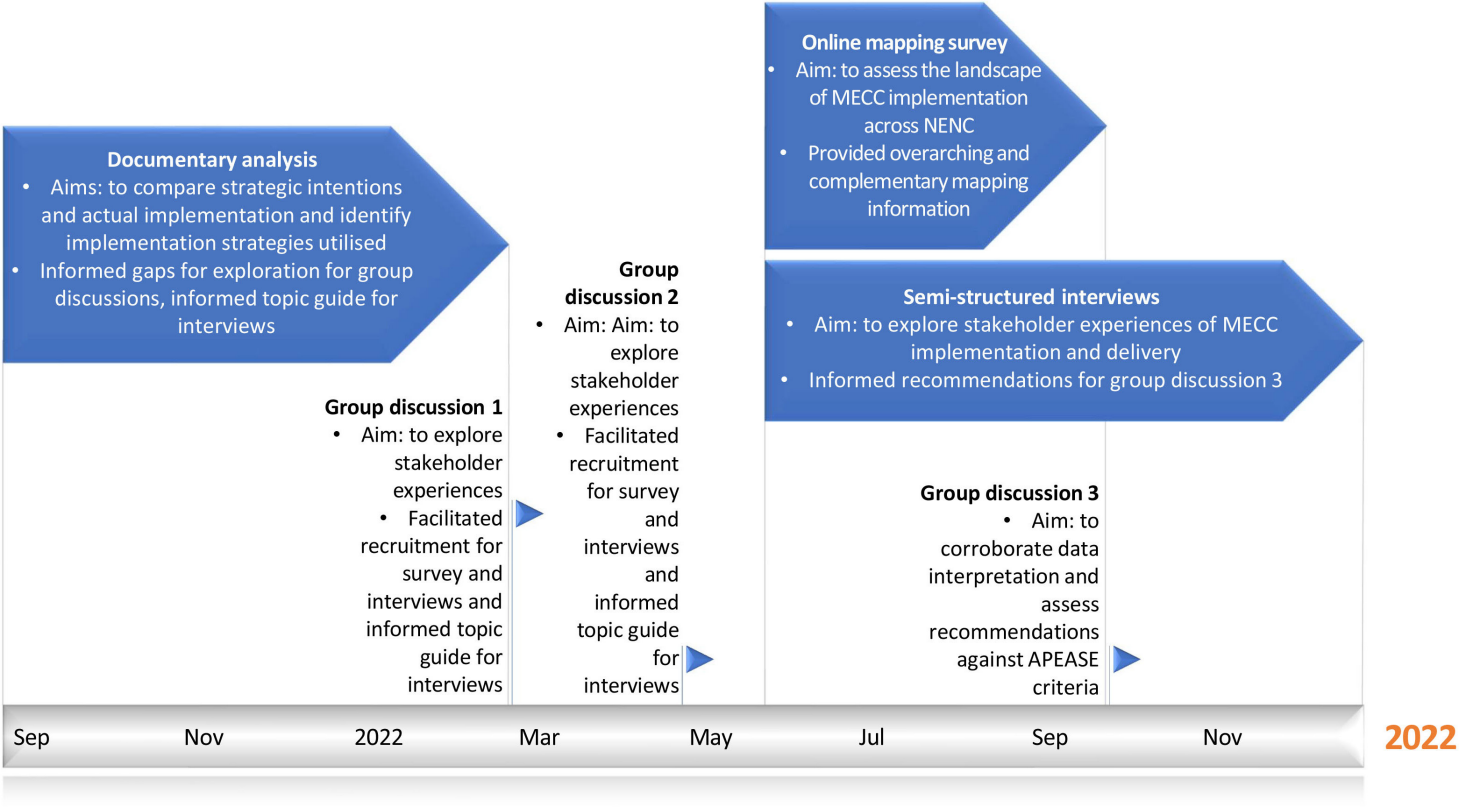
Timeline of data collection packages and the aims of each one. APEASE, affordable, practical, effective, acceptable, safe and equitable; MECC, Making Every Contact Count; NECC, North East and North Cumbria.

This multimethod, process evaluation applying NPT and TDF conducted between September 2021 and December 2022 included four work packages using document review, quantitative survey, qualitative research and stakeholder engagement (see figure 1 for the process of activities and their guiding frameworks and figure 2 for a timeline of events and aims of each activity). The protocol was preregistered and can be found on the Open Science Framework: osf.io/ fz436.^33^ This study and related findings are reported in line with the Standards for Reporting Implementation Studies checklist^34^ and specific guidance for mixed methods studies from the EQUATOR Network website.^35^

### Public involvement

At the development stage, the project proposal summary was circulated to the North East and North Cumbria (NENC) MECC regional Strategy Group mailing list for feedback. The group has 60+ members (including volunteers, service users and members of the public) and is made up of several smaller, locality-based MECC groups. Comments were received from 23 lay representatives (positive response; small changes/amendments made to objectives and outputs). During the project, two lay advisory groups (LAG) were arranged. One LAG was convened from the NENC MECC regional strategy group (n=6) and included representatives involved in the delivery, commissioning and implementation of MECC. The healthcare professionals LAG met three times throughout the project (October 2021, March 2022 and September 2022). Another LAG was arranged with members of public (n=2) and met three times (January, March and September 2022). This public engagement model provided an informal space to feed into project development, materials, outputs and dissemination (eg, lay summary). The project was awarded an ‘Investing in You—Dialogue & Change’ Award in recognition of the involvement of community members in dialogue that leads to change.

### Setting and participants

Set up in 2016 in response to a national focus on the prevention and public health agenda,^36^ the NENC MECC strategy group develops cross-sector policy and strategic plans to facilitate a joined up, consistent approach to MECC implementation and evaluation across the region. We worked with a key contact through the MECC strategy group (CR) to cascade the project activities to all organisations implementing MECC across NENC (ie, local government (13 in total across the region), healthcare and other public, private and VCS) via their mailing list. This included a diverse range of contexts including fire and rescue services, general practitioner practices, healthcare trusts, local government and VCS organisations (eg, foodbanks, community groups). Organisations were invited to take part in the various elements of the project (ie, document review, quantitative survey, qualitative research and stakeholder engagement). All participants provided informed consent to take part.

### Data collection and analysis

#### Triangulation of mixed methods

Analysis and reporting of findings triangulated qualitative and qualitative data through both qualitising (narrative synthesis) and quantitising (calculated frequencies for prioritisation of themes).^37^ All data sources were treated with equal importance, in line with a triangulation design.^38^

#### Documentary analysis

We reviewed and appraised documentation by systematically mapping written information supplied by local organisations implementing MECC (n=5 out of 28 organisations contacted) between September 2021 and July 2022. Relevant documents included local/regional MECC strategies, implementation plans, logic models/theory of change, programme updates (if available), evaluation plans and associated learning. Our systematic analysis approach included a focus on comparing strategic intentions and actual implementation, through comparison of data across the organisations that supplied documents. Data extraction (online supplemental additional file 1) included implementation strategies (Expert Recommendations for Implementing Change (ERIC) taxonomy),^39^ behaviour change techniques (BCTs taxonomy v1), MECC Implementation guide and Quality Markers for Training^40^ and NPT constructs.^41^ The use of the NPT coding manual facilitated a systematic data analysis process while allowing for the generation of codes and themes throughout analysis.

Data credibility was achieved by including input from two researchers (AMR and BG) to verify the initial coding conducted by CC. This involved AMR, BG and CC separately coding documents with any differences in the coding resolved through discussion. A subset of the documentary analysis template was also checked by TF—an expert in implementation science and NPT. This part of the study was also informed by high-level implementation data collected by the MECC coordinator (CR) (retrieved from the local integrated care system training app) and collated in an anonymous overview document shared with the research team. This high-level implementation data included details on the following: stage of implementation, liaison meeting, existence of a logic model and/or implementation plan, and training delivery (the number of attendees/date of delivery). Documentary analysis offered a starting point for the content and structure of the group discussions by highlighting gaps for further exploration.

#### Online survey

All stakeholders who signed up for the MECC strategy group mailing list (n=86 stakeholders) were invited to take part in the online survey via email invitations from the MECC Strategy group between June 2022 and September 2022. The mailing list includes representatives from the following sectors: local authorities/government, NHS healthcare trusts, emergency services, NHS integrated care board managers and academics. The online survey provided initial, high-level baseline data on the current MECC implementation landscape across NENC (n=34 out of 86 approached). The survey was hosted by Qualtrics (online survey tool) and took approximately 15–20 min to complete.

The survey (online supplemental additional file 2) gathered data from organisational MECC Leads on implementation timeframe, stages of implementation (planning/education/training/delivery/evaluation), staff groups reached, client groups reached, relationships with other organisations, training structure, nature of MECC contacts with service users, numbers of staff/service users involved, COVID-19 impact, Capability, Opportunity, Motivation and Behaviour (COM-B) items^42^ and a validated instrument (Normalisation MeAsure Development questionnaire (NoMAD)) for measuring implementation based on NPT.^43^ Due to the survey being anonymous, the number of organisations which took part within this phase was not collected. The survey data were analysed by using SPSS V.26 to generate descriptive statistics. Due to a lack of data, survey findings were not presented in isolation. Instead, descriptive statistics from the survey data were triangulated with the remaining data narratively to inform findings.

#### Qualitative interviews and group discussions

All stakeholders who signed up for the MECC strategy group mailing list (n=86) were invited to take part in the interviews and group discussions via email invitations from the MECC Strategy group between March 2022 and December 2022. During the first and second group discussions (held virtually, via Microsoft Teams), Padlet, an online interactive platform designed to simulate a virtual notice board, was used to facilitate discussion and document written responses. The interactive platform provided a series of questions based on the group discussion topic guide which aimed to prompt responses around planning and implementation, service reach, relationships with other organisations and interventions delivered. Responses received were used to facilitate further discussion on the topics explored within the first two group discussions and were integrated with the qualitative data from the group discussions and interviews to inform themes. Due to low participation within the first and second group discussions, data were not collected about participant’s settings. The third group discussion (held in October 2022 in person, at the university) first presented the initial headline findings from the project which included some of the interviewees to check whether the data interpretation matched their understanding of MECC implementation, providing an opportunity for further data confirmation through discussion. Next, stakeholders were asked to work in groups with other stakeholders from the same setting (local authority or healthcare) to cocreate an implementation plan and logic model using the NENC templates. Also, stakeholders were asked to evaluate the recommendations created from the project findings (more information on this activity is available under ‘Development of recommendations to support future implementation’). After both activities, all stakeholders convened to provide their feedback and engage in discussion. Purposive sampling was used to obtain a spread of experience across locality, setting (eg, NHS vs local government) and stages of implementation (starting vs established implementation). Attendees included representatives from local government (n=9), NHS settings (n=6), VCS (n=3) and higher education settings (n=1), with 17 organisations across NENC represented. We did not collect information on the specific organisations represented by NENC stakeholders during workshops 1 and 2.

As above, members of the MECC strategy group mailing list were invited to take part in one-to-one interviews via email invitations. Recruitment was targeted to collect a purposive sample, again within setting and the stage of implementation. As all members of the MECC strategy mailing list were invited to take part in both the group discussions and the interviews, 5 participants took part in both aspects of the study, with 13 organisations across the NENC represented in total. Interviews were conducted by (CC=17) and (AMR=1) online via Microsoft Teams, with interviews ranging between 35 min to 1 hour 30 min in length. Topics addressed within the interview topic guide aimed to elicit responses on the participant’s role in MECC implementation, what MECC means to them, recalling early implementation, motivations for implementation, acceptability of MECC, support and resources, the influence of other organisations, current stage of implementation, impact and outcomes of MECC, and how implementation could be improved. Topic guides were informed by NPT and TDF and were iteratively piloted and developed in response to feedback from early participant interaction (online supplemental additional file 3). All group discussions and interviews were digitally audio recorded, transcribed verbatim and analysed using the framework method.^44^ Early findings were used to inform later interviews and the third group discussion, with the aim of achieving thematic comprehensiveness.

Qualitative data were collected, coded and checked by CC, AMR, BN and BG with use of NVivo V.11^45^ to facilitate data management. The data trustworthiness was achieved by including interview data from the perspectives of a range of stakeholders (eg, NHS, local government, delivery and leadership staff). Input from three researchers (AMR, BN and BG, experienced qualitative researchers) was used to verify the initial data interpretation conducted by CC. This involved AMR, BN, BG and CC separately reviewing interview transcripts with any differences in the coding resolved through discussion. The document review, survey data and qualitative data were analysed in isolation and then the findings compared with interpret to what extent they converged, diverged or complemented one another.

We employed an inductive thematic analysis method, allowing themes to emerge from the data, a common practice in TDF studies. To analyse the interview transcripts and identify themes, we used the framework method.^46^ The transcripts underwent multiple readings by researchers (CC, BN, BG and AMR) and were meticulously coded line by line to discern patterns. After an initial inductive coding of a subset transcripts (n=3), a coding framework was created and mapped onto NPT and TDF domains. Subsequent analysis of all transcripts was deductive according to the created coding framework, with adjustments made as necessary.

To identify key TDF and NPT domains influencing the implementation of MECC, themes were prioritised in relation to the number of transcripts that contained the domains (as an indicator of relevance), the number of themes generated within each domain (as an indicator of elaboration), triangulation between subthemes across interviews and group discussions within each domain and level of agreement with NPT domains derived from NoMAD survey items (as corroborators of findings) and conflict within domains (as an indicator of coherence). Using standard criteria and considering these factors together allowed domains to be identified as key domains within the data.^47^,^50^

To explore the relevance and influence of each TDF/NPT domain to MECC implementation, domains were mapped in terms of the number of participants coded for each and whether each was a barrier, facilitator or contained a mixture of both and displayed in table format. The data from the third group discussion were used to support and supplement the themes developed from stakeholder interviews.

#### Development of recommendations to support future implementation

The study team used the findings from the qualitative interviews and documentary analysis to draft a list of practical recommendations to strengthen implementation of MECC. These recommendations included examples to improve implementation strategies. To enhance the suitability and acceptability of these recommendations, the third group discussion was conducted by collecting data from 19 stakeholders (interviewees) (total of 85 min). Stakeholders independently rated whether each recommendation was affordable, practical, effective, acceptable, safe and equitable criteria,^51^ on a dichotomous scale of yes (1), no/uncertain (0) for each criteria. This gave a total possible score of 114 for each recommendation (19 stakeholders×6 points max per recommendation). Immediately after individual ratings, we encouraged discussion surrounding uncertainties and potential modifications to recommendations during a collaborative, group discussion. The third group discussion feedback was incorporated into a refined recommendations table, which was then presented to the NENC MECC strategy group. This process resulted in the final list of recommendations presented in this paper.

### Findings

#### Triangulation of mixed methods

Given the relatively low response rate (18% for documentary analysis; 22% for complete survey data) and overlap between content, findings from the documentary analysis and online survey were combined, although the sources of data to inform each subheading below are labelled. The majority of stakeholders reported difficulty in creating and locating documents due to staff turnover. Documentation was received from a total of five organisations and, despite several invitations, no further updates to map implementation progress were received. Logic models were provided by two organisations and one organisation provided an implementation plan relating to MECC. Pretraining and post-training feedback, and MECC strategy and programme updates were received from two organisations, and training slides, a business plan and an induction checklist by one. An overview of the document analysis is provided in online supplemental additional file 1. Examining the quantitative survey data, 34 participants partially completed the survey and 19 participants had complete data for all survey items, results of which are displayed in onlinesupplemental additional files 2  4. Only three participants responded that their organisation was involved in MECC delivery indicating a lack of representation of patient-facing roles, perhaps explaining the low response rate for survey items related to MECC delivery. Individual, semistructured interviews (n=18, 4 male, 14 female, across 13 organisations) were conducted remotely, between June and December 2022. Interviews lasted an average of 69 min (range 36–108 min). 10 participants worked within a council, 7 within healthcare settings and 1 as a regional MECC coordinator. Interview participants were marked with anonymous identifiers P1 to P18. Three interactive group discussions aimed to explore stakeholder experiences (n=48; 225 min) and were delivered through the MECC regional strategy meetings (March 2022 (30 min; 19 participants); May 2022 (120 min; 10 participants) and October 2022 (75 min; 19 participants)). Group discussion participants were labelled according to the group discussion the quotations were from (GD1 to GD3).

#### Stage of MECC implementation: findings from the document analysis and survey

Stage of implementation was generally early, indicated by both document analysis and survey results. A document was provided by the MECC coordinator (CR) highlighting an overview of implementation across the region. Of 28 organisations included, 5 had not yet attended an initial meeting with the MECC coordinator. For the 19 organisations where stage of implementation was self-reported by survey participants, no organisations had reached the most advanced stage where MECC was fully embedded within the organisation and was part of the long-term plan. Planning was also the most common implementation stage of the remaining documents (5/5 organisations), although almost all included documents showed evidence of pretraining and post-training evaluation (4/5 organisations). Most survey participants reported being involved in implementing (n=20) with some also involved in delivering MECC (n=14), and participants had been doing so for a reasonably short amount of time (mode=1–5 years). Perhaps relatedly, few survey participants were involved in MECC (mode=0–10 staff) and communication around the importance of MECC was needed, as demonstrated from responses to the NoMAD items in online supplemental additional file 4. From the overview document supplied by the MECC coordinator (CR), nine organisations were working towards short to medium targets (ie, using refreshed MECC content and active trainers delivering local and regional priorities), six were still forming the content of an implementation plan and logic model and two were at the beginning of their MECC journey—in the process of reviewing MECC by assessing local priorities.

#### Implementation strategies: findings from the document analysis and interviews

From the documentary analysis, the MECC implementation packages included various strategies designed to change behaviour at organisational, practitioner or patient levels. A total of four BCTs were identified (adding objects to the environment, prompts/cues, self-monitoring of behaviour and social support: unspecified). Only one BCT was used by more than one document, namely adding objects to the environment, which was used by two documents (2/5 organisations). Details of implementation strategies as aligned with the ERIC taxonomy can be found in table 1, with application examples provided from the interview data. The number of ERIC strategies used varied between 18 and 42 and the number of quality markers for training ranged between 3 and 10. Implementation strategies used within more than three documents were as follows: ‘create a learning collaborative’ (3/5 organisations), ‘assess for readiness and identify barriers and facilitators’ (2/5 organisations), ‘build a coalition’ (2/5 organisations), ‘conduct educational meetings’ (2/5 organisations), ‘develop educational materials’ (3/5 organisations), ‘distribute educational materials’ (3/5 organisations), ‘promote network weaving’ (2/5 organisations), ‘purposely examine implementation’ (2/5 organisations), ‘stage implementation scale up’ (3/5 organisations) and ‘use train-the-trainer strategies’ (3/5 organisations). Although mostly at an early stage of implementation, some training delivery was reported between April and December 2022. Results of the MECC overview document showed that 46 train the trainer sessions were recorded, mainly driven by NHS foundation trusts and local government, resulting in a total of 345 active trainers, with 8 being the maximum number of sessions held by any one organisation. There was a lack of information within the document analysis regarding topic areas of training, most commonly delivered by primary healthcare professionals or NHS specialists (3/5 organisations) and across the community (2/5 organisations). One of the lasting impacts of COVID-19 reported included a shift towards online training, with survey participants reporting an equal experience of face-to-face and online training.

**Table 1:** Implementation strategies with applied examples mapped onto the ERIC taxonomy

NPT; TDF	Implementation strategy (as mapped onto the ERIC taxonomy)	Application to MECC implementation/delivery
Cognitive participation; behavioural regulation	Involve executive boards	Gain buy-in from senior leadership and executive boards
	Mandate change	Executive board identify MECC as a priority, either specifically or as part of a wider agenda, for example, prevention
	Purposely re-examine the implementation	Improved and standardised evaluation strategies needed to assess implementation. Standardised implementation strategy needed
	Stage implementation scale up	Use of pilots within organisations to trial MECC implementation and gather evidence of positive outcomes
Collective action/cognitive participation; environmental context and resources	Develop educational materials	Development of MECC resources by MECC coordinator/steering group, with the option to tailor
Collective action; skills	Make training dynamic	Allow for training to be tailored to fit with the setting/organisation
	Promote adaptability	Allow for tailoring of MECC resources while retaining consistency over the key MECC message
	Use train-the-trainer strategies	Continue train the trainer model, but apply one or more of the following; Assign MECC ‘champions’/health ‘advocates’/health coaches Buddy-up systems Peer support groups Selecting only ‘confident’ people to receive train the trainer training Setting cascade expectations at sign-up stage
Collective action; social influence	Distribute educational materials	Improve communication with the MECC coordinator/steering group to raise awareness of existing available materials and allow them to be disseminated
	Promote network weaving	Continued use of MECC steering/strategy group to promote information sharing, support and shared learning
	Work with educational institutions	Continue train the trainer model to provide MECC training at scale
	Use advisory boards and workgroups	Use of MECC steering/strategy group to develop core materials and build and communicate a consensus understanding and branding of MECC

ERICExpert Recommendations for Implementing Change strategiesMECCMaking Every Contact CountNPTnormalisation process theoryTDFtheoretical domains framework

P5: ‘so really early on the MECC training was delivered face to face. We then launched our strategy and encouraged MECC training and then it had turned to be online, so the offer had changed quite slightly’

#### Enablers and barriers to MECC implementation: all sources of data

##### Key domains of influence

From the qualitative data, 10 key theoretical domains influencing MECC implementation were identified: ‘behavioural regulation’ (cognitive participation), ‘environmental context and resources’ (collective action/cognitive participation), ‘skills’ (collective action), ‘goals’ (reflexive monitoring), ‘social professional role and identity’ (cognitive participation), ‘beliefs about capabilities’ (cognitive participation/collective action), ‘social influences’ (collective action), ‘beliefs about consequences’ (reflexive monitoring), ‘knowledge’ (coherence) and ‘reinforcement’ (cognitive participation). There was generally a wide spread of subthemes across domains and evidence of conflicting statements within domains (eg, if some participants report lack of confidence to have MECC conversations whereas others report having confidence). Table 2 provides an overview of the prioritisation of themes across participant interviews and group discussions, as categorised by the TDF and NPT domains. Themes, subthemes, and example codes and corresponding quotes derived from participants interviews and group discussion are displayed in online supplemental additional file 5.

**Table 2:** Prioritisation of the TDF domains (mapped onto the four NPT domains) from qualitative data in terms of frequency, number of participants elaborating on them, and conflicting beliefs within each domain

Ranking	NPT; TDF domains	Number of participants coded for domain	Number of themes within each domain	Triangulation (interview subthemes)	NoMAD domains (agreement)	Conflict within domains
1	Cognitive participation; behavioural regulation	18	4	Yes	First	Yes (mixture of top-down and bottom-up processes used and favoured, variation of embedment of MECC within organisational processes, mixed use of implementation documents)
2	Collective action/cognitive participation; environmental context and resources	18	6	Yes	Second	Yes (mixed availability of regional/national MECC resources and funding for staff capacity to implement and deliver training, COVID-19 exerted both positive and negative effects on MECC implementation)
3	Collective action; skills	18	1	Yes	Second	No
4	Reflexive monitoring; goals	18	2	Yes	Third	No
5	Cognitive participation; Social/professional role and identity	18	2	No	First	Yes (a minority reported an initial lack of interest in MECC, variation in whether there is sufficient accountability for MECC role within organisation)
6	Cognitive participation/collective action; beliefs about capabilities	16	2	No	First	No
7	Collective action; social influence	18	2	Yes	Second	Yes (common use of networking but little use of involvement of service users)
8	Reflexive monitoring: beliefs about consequences	18	6	No	third	Yes (consensus of the potential of MECC but some uncertainty of actual effect due to variation in the process of implementation)
9	Coherence; knowledge	18	4	No	Fourth	Yes (varying prior knowledge of MECC, more knowledge of resources and organisational fit than of evidence to support MECC)
10	Cognitive participation; reinforcement	12	2	No	First	YES (MECC cited as intrinsically rewarding to deliver but financial incentives were rare)

MECCMaking Every Contact CountNoMADNormalisation MeAsure Development questionnaireNPTnormalisation process theoryTDFtheoretical domains framework

12 of the 26 identified themes during participant interviews were also identified during the group discussions. The subthemes that were pertinent across both interviews and group discussions were the importance of a top-down approach, challenges of recording the outcomes of MECC, challenges of COVID-19 to MECC implementation, the importance of networking for shared knowledge, resources and support, the importance of tailoring for training and resources, the cascade model is favoured but implementation often halts at train the trainer and that MECC helps achieve wider organisational goals. Subthemes that were more frequently mentioned related to the implementation process, knowledge needed to implement/ deliver MECC, roles, responsibilities and accountability, feeling of involvement with MECC, maintenance of MECC in organisation, recording/ monitoring MECC, networking with other organisations, materials/resources, staff capacity, training and goals that align with MECC implementation. Within implementation process, the most commonly cited process was a top-down approach. This was cited by participants as the most common but also most likely to be valued by participants in comparison to a bottom-up approach.

P12: ‘the fact that’s it embedded in their portfolio and their job description or whatever, so they are assessed on it within their appraisal process, that helps immensely. But that would do nothing if you didn’t have the support of the directorate or the team that you’re embedded in’

##### Enablers to MECC implementation

When maintaining MECC within an organisation, the most important factor was maintaining buy-in, particularly from leadership and chief executives, reflecting the previous theme. The most essential knowledge needed to implement MECC, as revealed by our qualitative data sources, encompassed an understanding of the organisation itself, insights into how MECC could be effectively implemented within its specific context and strategies for building successful partnerships with other organisations. Feedback on MECC materials and resources (eg, signposting tools) was generally positive and many participants noted their considerable improvement, although many also tailored resources to fit their organisation or setting. Qualitative findings highlighted that partnership and networking were most notably externally, between organisations. Many participants felt an affiliation with MECC that extended outside of their professional role, although implementation was further facilitated by creating clear roles and accountabilities relating to MECC implementation within organisations. A final key facilitator of MECC implementation indicated in the qualitative data were the belief that it helped to achieve a range of goals that the organisation aimed to achieve, both goals specific to the organisation and to encourage wider scale change. While beliefs in MECC as a means of achieving a range of goals within participant’s organisations and for partnership working, the low number of documents provided for analysis and an observed limitation to discussion of logic models for MECC implementation in the group discussions, suggest that it may be both challenging, but an opportunity to improve MECC implementation through supporting engagement with planning tools.

W1: ‘MECC supports key areas such as the Joint Health and Wellbeing Strategy’

Demonstrated by the survey data, self-perceived knowledge, skills, resources and support appear to be a key enabler, as recorded by the COM-B survey items (online supplemental additional file 4). Subsequently, most survey participants reported not requiring any further support. However, sharing of knowledge and information was greatly valued. From the survey dataset, belief in the effectiveness of MECC is also indicated as an enabler of the embedding of MECC. This belief varied widely, from some holding a strong belief of effectiveness even without the availability of formal evidence, to others who were highly motivated to implement and deliver MECC and less concerned about effectiveness. Within the survey data, MECC conversations were generally reported to happen, varying from individual and group delivery, and face to face and online.

P6: ‘budgets are getting cut and there’s less money within the system to be able to have public health programmes, so if we’re really going to make a change then it is about some for those MECC conversations being able to happen’

##### Barriers to MECC implementation

The most pertinent barrier to the implementation and delivery of MECC was a difficulty measuring the impact of MECC, due to its brief nature and low likelihood of follow-up with the service user. This is related to the previous two subthemes as leadership often asked for evidence of impact, which was difficult to provide. Within the most commonly cited theme of materials and resources (eg, staff, implementation plans and funding), the most pertinent subthemes were the issue of staff capacity, both due to competing workload priorities of the person implementing MECC, and the time for staff to attend MECC training. Related, COVID-19 further negatively impacted staff capacity as efforts were directed towards the COVID-19 response. Additionally, survey data highlighted other lasting impacts of COVID-19 including MECC no longer being prioritised, possibly reflected by the findings that only around half of participants’ time was dedicated to MECC.

P10 ‘I think that is something actually that we lack, and we’ve talked about it in our team of like my team, how do we measure the success of MECC because it’s really hard. Because it’s brief intervention they’re gone, and you might not see that person again. Some people will and we can develop those case studies, but in most instances the point of it is, isn’t it, that you don’t need to follow up with that person’

Although most participants considered the cascade train the trainer model as favourable to implement MECC at scale, the most frequently cited barrier was cascade ending at train the trainer. Interviewees provided many suggestions to address this barrier, included in table 1.

P17: ‘But it’s not to the level of where they are then cascading it to their team… when we’ve looked at train the trainer models that’s where we fell down really’

Finally, the qualitative data also indicated that there may not be high coherence (NPT) about what MECC looks like and means in practice. Participants’ accounts of MECC indicated that the definition and meaning of MECC varied, from some comparing MECC with very brief and brief interventions, to other describing MECC as an approach underpinning interactions. There was some agreement that MECC is opportunistic and person-centred, although there lacked consistency of what the content of MECC conversations should be, such as motivational interviewing or signposting.

### Development of recommendations to support future implementation: findings from group discussion 3

Qualitative findings suggested difficulties in developing logic models and implementation plans (subtheme: Implementation process and resources);

P9: ‘we probably did initially and I think it probably just went by the wayside over time as, erm you know, people moved into different roles and other people took over and I think by the time I started in (name of location) there wasn’t really a formal process in place as such, and I think you know again it’s one of those things that we probably like to have and would be good to have, but just because of capacity and other priorities it just hasn’t happened, isn’t in place just yet’

Consequently, the third group discussion offered the opportunity to develop logic models for MECC implementation. Given the differences in implementation pathways, logic models were developed and collated separately for healthcare and local government. Example logic models were collated from eight logic models within local government and the VCS sector, and two from healthcare settings. Stakeholders were also asked to complete implementation plans during the third group discussion, but as responses to these were minimal and incomplete, data were instead used to inform the template logic models. Onlinesupplemental figures 1  2 provide the collated logic models for local government/VCS and healthcare settings, respectively. Similar concepts were grouped and the frequency of occurrence of each element on the contributing logic model and implementation plans created by stakeholders is displayed in brackets in figures1  2. One difference between the logic models is that the NHS model places a higher emphasis on informing the executive board and senior management throughout the implementation process, whereas the local government model only cites top-down buy-in as needed for inputs. The NHS logic model also places less emphasis on training and more on implementation process.

In the third group discussion, discussions about the recommendations that had been developed from the preceding data collection (see online supplemental additional file 6) showed variability in endorsement among group discussion participants. These recommendations included examples to improve implementation strategies currently being used such as investing in staff resources to increase staff capacity and creating a supportive mechanism to support training cascade.

W3: ‘If you’ve got a lead with buy-in from senior sources, you’ve got champions for supporting training and peer support, you’re building an infrastructure and culture’

Scores for each recommendation ranged from 55 to 85 (median=71) (online supplemental additional file 7). None of the criteria for any of the recommendations reached a full consensus (see online supplemental additional file 6), although the recommendations around support for cascading, online training, increasing buy-in and increasing accessibility of resources received the highest acceptability scores. Recommendations around increasing staff resource and appointing an MECC lead within the organisation received the lowest scores. Combined with the influence of discussion during the third group discussion, only two recommendations remained the same, two were merged into one recommendation and a recommendation specific to research was added. The remaining recommendations were amended (table 3) and provided the overall output of the study.

**Table 3:** Amended recommendations following discussion with stakeholders during workshop 3

Recommendation	Example delivery
Create a standardised infrastructure and strategy to combat delivery/implementation issues	Producing a ‘living’ logic model to improve efficiency of implementation, particularly for staff handoversA hierarchical structure of MECC leads and MECC champions
Ensure buy-in from senior management to facilitate a change in organisational culture	Set up steering groups, ensure MECC is a standardised item on team meeting agendas, organise a credible source to talk about MECC at the organisation
Encourage further ‘buy-in’ of MECC from additional pathways/organisational departments by disseminating evidence of effectiveness of the programme	Sharing case studies that are relevant and applicable to trainees to demonstrate understanding of their role
Create a support system and expectations around cascading and make these explicit at sign-up and recruitment stages of MECC cascading training	Offering a support package after cascade training for example, monthly peer support meetings and topic-specific sessionsInclude cascade expectations (eg, 4 sessions over 12 months) on MECC cascade training flyer
MERGED: Allow for tailoring of training (hybrid model of online and face to face) and resources to attain organisational fit without losing the consistency of the MECC message. Provide short e-learning on how to use and tailor these resources	Core MECC training available in face to face, online, or self-paced formats, and core training resources available to amend according to the organisation, setting and occupation
Remained the same but acknowledged to be a long-term goal	MECC as part of staff inductions and return to work interviews
Create a consensus on what data to collect around MECC implementation and delivery across the region and embed within current systems, with allowance for organisational tailoring	Agree on core items to measure during regional MECC strategy groupAdding a ‘delivered MECC’ tab to existing internal recording systems
Consider how organistional and setting specific case studies could be developed to provide evidence of optimisation of implementation	Drawing on setting and organisational specific case studies to both present to senior management and use for training
(Research) Explore the effectiveness of MECC on the outcomes of service users using both quantitative and qualitative methods	Randomised control trial comparing MECC conversations with usual care control, with a process evaluation

MECCMaking Every Contact Count

## Discussion

### Summary of key findings

Overall, there was a consensus that MECC is an opportunistic and universal approach to health behaviour change. The current study finds that MECC implementation is mainly in its infancy across the region, despite strong leadership, supportive infrastructures and top-down support. A strong belief across stakeholders was that no evidence of the effectiveness of MECC was needed to incorporate it into practice. This may demonstrate the difference in perceptions of MECC as an approach rather than an intervention.

Our findings revealed a range of 18–42 implementation strategies^39^ used across documents, with a notable emphasis on educational and training approaches. This aligns with literature highlighting an over-reliance on education-based implementation strategies.^52 53^ However, the range of 18–42 strategies appears consistent with typical numbers reported in implementation studies, often falling between 16 and 59 strategies depending on project goals and scale.^54^,^57^

Ten TDF domains (and respective NPT domains) were identified as influences for the implementation process of MECC. In particular, six subthemes had substantial support across the qualitative data, including the importance of a top-down approach, challenges of recording the outcomes of MECC, challenges of COVID-19 to MECC implementation, the importance of networking for shared knowledge, resources and support, the importance of tailoring for training and resources, the cascade model is favoured but implementation often halts at train the trainer, and that MECC helps achieve wider organisational goals.

Partnership and networking were seen as key to providing support and shared learning, including the use of steering and support groups, with the MECC regional coordinator as central. Metz *et al*^58^ have recently modelled the importance of building trusting relationships in the implementation process, as key to achieving desired implementation outcomes. Our work here on implementation of MECC—which particularly relies on interagency and cross-sector working—further highlights the establishment and maintenance of trusted partnerships as a key implementation enabler. Partnership was mostly between organisations rather than involvement of service user groups. Perhaps also related to an early stage of implementation, a standardised MECC brand and implementation strategy was important. This ‘relational restructuring’ (NPT construct) that changed the ways people are organised and relate to each other^41 59^ was crucial to support sustainability of the MECC implementation. The threats to sustainability included staff shortages and turnover and the resultant loss of MECC expertise, in addition to potential competing demands from other safety initiatives. As implementation was often delayed due to staff turnover and familiarisation with the progress of implementation for the new staff, the use of updated logic models could help to address this barrier. As such, the logic models within this paper provide some guidance as a template, and two models reflect the frequent separation between local government and healthcare implementation processes. For implementation research, Smith *et al*^60^ offer a logic model template that is designed to facilitate planning and delivery of implementation projects. Our findings indicate that implementers in healthcare and local government settings may find such logic models challenging to develop, but with guidance, helpful for planning and ongoing implementation of MECC within their organisations. The logic models we have developed with workshop participants are an immediately useable output of this research for those implementing MECC. The sustainability of MECC, our findings suggest, required changes in professional relationships and communication (‘relational restructuring’) to accommodate altered workflows and successfully facilitate cross-regional/sector collaboration and the sharing of MECC expertise and knowledge.

The current study found MECC implementation to be driven by a few influential people, most commonly through top-down buy-in such as support from management and leadership and chief executives. The main mechanism of the top-down approach was cascade training, although barriers including staff capacity for training were identified along with suggested solutions. Given the dominance of the top-down approach, MECC implementation was powerfully facilitated by organisational goals that aligned with MECC implementation. However, implementation was also stimulated in a bottom-up way from a small number of invested individuals where MECC is within their role. Namely, most staff were passionate and motivated to implement MECC, both within and outside of their role responsibilities, despite the lack of normative evidence of improved service user outcomes. Evidence of effectiveness was more important in achieving top-down buy-in. The role of such ‘champions’ in successful implementation is much discussed in the literature, with caution about over-reliance on specific individuals when trying to achieve system-level change.^61^ Future work is needed to create a wider organisational change that expands beyond the small number of individuals where MECC is within their role. In accordance with the ERIC taxonomy,^39^ this may involve the following strategies: ‘revise professional roles’, ‘conduct ongoing training’ and ‘conduct educational meetings’.

Concerning training and resources, tailoring and flexibility were key while retaining a consistent MECC brand and message. A hybrid model of online and face-to-face training and resources was favoured, stimulated by the COVID-19 pandemic. However, while adapting content helped to retain relevance for a wide variety of organisations and settings, it became more difficult within training of a wide range of staff groups and organisations.

Maintenance of training cascade after train the trainer was highlighted as a challenge by stakeholders. Previous literature identifies similar challenges in cascade training models, mostly linked with insufficient confidence and organisational challenges.^62^,^64^ Some of these challenges are linked with unanticipated organisational restructuring, resource limitations and lack of opportunities to develop local training partnerships to support roll-out. Theoretical explanations of behaviour change maintenance^65^ provide suggestions on how to facilitate behaviour change, including fostering positive maintenance motives, facilitating behaviour self-regulation; facilitating habit development; providing individuals with resources (physical and psychological) and providing social support and introducing social changes. Coincidingly, potential solutions identified by stakeholders could include making expectations for cascading clear at the sign-up stage of training, encouraging ‘buddy-up’ collaborations for training delivery and creating peer support groups for trainers.

The need for high-level, strategic commitment to ensure the long-term success of MECC emerged as a key theme in this study, mirroring findings elsewhere in the literature.^22 24^ This commitment supports the need to regularly refresh and reinforce key public health messages responsible for driving MECC forward in the longer term and the wider capacity required to support it locally. Linked to this need, although there was a general consensus of MECC as opportunistic, there was uncertainty and disagreement about MECC as an intervention or approach, indicating low coherence and levels of collective ‘sensemaking’,^30 31^ supporting theoretical explanations from implementation science (ie, NPT) about conditions for successful implementation. The definition and meaning of MECC varied, from some comparing MECC with very brief and brief interventions, to other describing MECC as an approach underpinning interactions. This dichotomy perhaps relates to the variation in beliefs around measurement of and reliance on evidence of effectiveness for implementation, outlined later in this section. This suggests the need for an updated consensus definition. Subsequently, a Delphi study, currently underway, will help to reach an agreement over what MECC describes, facilitating the delivery of consistent and clear information about MECC.^66^

There was a complete lack of any form of patient and public involvement (PPI) into the MECC implementation processes described, perhaps reflecting a lack of understanding about what PPI entails. Understanding lived experience is essential when looking at the dynamic and evolving relationships between actors within a system.^67^ Education on how to meaningfully incorporate PPI is needed to facilitate bottom-up change^67^ and a potential strategy could be to involve experts with experience in the implementation process.^39^ Langley *et al*^68^ propose three domains of influence when people from different sectors come together to engage in creative play: influence on the process of implementation (the intervention generated through creative play is ‘owned’ by end-users; the intervention incorporates research, experiential and contextual knowledge and comes with the testimony of end-users who were involved in the making; it includes a ‘boundary object’ in physical or visual form that acts to engage others beyond the codesign group; and it typically includes ‘core’ and ‘adaptable’ elements). There are challenges in involving PPI in scenarios where it involves more high-level processes (eg, policy implementation), but the key should be on how to find ways around this.^69^

### Recommendations for practice and policy

The findings from this analysis indicate a number of key areas for further uptake and roll-out of MECC. The implementation of MECC could be further supported by creating a shared architecture including infrastructural and strategic resources to collaboratively support delivery/implementation issues. Two examples of this from our study might be salutary first the regional MECC team’s attempt to create a shared service directory (via the MECC Portal). Second, drawing on the logic model activities in the workshops to produce a ‘living’ logic model to improve efficiency of implementation, particularly for staff handovers. The logic model templates cocreated with stakeholders as part of this project potentially provide a resource for organisations at an early stage of implementation that could be customisable to fit a specific organisation and local needs. These activities highlight the importance of developing implementation practitioner competencies and skills, such as knowledge about the intervention/context, support change processes and facilitate evidence-based practice,^70 71^ and conducting comprehensive context assessments, especially in early implementation phases.^72^

Recording and monitoring MECC implementation/delivery could be improved by creating a consensus on what data to collect about MECC implementation and delivery. This is important on both a practical level, and as guided by implementation theory, as such data are necessary for participants in the implementation process to engage in ‘reflexive monitoring’, which is needed to sustain engagement and commitment to the process of MECC implementation.^30^ The data collection would need to fit specific, organisational systems, but some core elements could be universal and aligned with the 3As^73^ elements (ie, Ask, Assist, Act). Such an approach could thereby provide input into wider regional system-level conversations around social determinants of health potentially scaffolded by conceptual frameworks such as Learning Health Systems geared to continuous improvement.^74^

To support sustainability and further support local organisational implementation, our findings provide evidence on the key roles/resources/relationships (or soft infrastructures) needed to be put in place at a regional systems level. These roles/resources/relationships will support more dynamic localised implementation activity moving beyond the current simplistic linear model of ‘share and spread’ and adoption mindset which fails to address the complex challenges of innovations within healthcare. Of relevance are the leadership of the MECC regional coordinator and the creation of a regional strategy group (networks), the online resources offered by the MECC portal, and the numerous networking opportunities available within the regular meetings set up by the MECC regional strategy group.

### Strengths and limitations

The MECC regional strategy group played a pivotal role acting in an advisory capacity, as a project steering group and study participant (interview), alongside with disseminating early findings. Through the MECC strategy group, we were able to share emerging findings, confirm the correct interpretation of findings and discuss ideas for upcoming stages, including feedback on draft recommendations. Our findings were used to formulate recommendations that were supplied to the MECC regional strategy group. From these recommendations, relevant stakeholders can make a strategic, informed decision using evidence-based recommendations to optimise the implementation of MECC and inform future research. Overall, the systematic approach taken throughout the present research and use of established theoretical frameworks, results in evidence which, importantly, facilitates efficient translation to policy and practice. This study combined relevant theoretical frameworks to understand implementation processes and provide explanations of behaviour at both group and individual levels and their interaction. Notably, our work also contributes to the developing area of bringing together barriers and enablers to inform implementation strategies.^75^ While mapping implementation strategies to barriers remains a challenge lacking consensus,^75^ our work provides an empirical example of linking implementation strategies to the identified contextual factors specific to optimising MECC implementation in our regional setting.

Despite its strengths, there are two main limitations of the findings reported here. First, there were significant recruitment challenges which resulted in a smaller than anticipated quantity of data for the document analysis and online survey. As such, the document analysis and online survey results mainly provide a description of general characteristics of some of the organisations involved in MECC across the region and are not fully representative of the wide variety of stakeholders involved in delivering/implementing MECC across the region. Furthermore, for the purpose of anonymity of survey participants, it is uncertain how many organisations the participants represented, thus assumptions that can be made around the generalisability to organisations across the NENC are limited. Also, although many of the survey questions were tailored towards the delivery of MECC, most survey participants were involved in MECC implementation rather than delivery, limiting the relevance of the survey for participants so that questions around MECC delivery remain unanswered. Thus, future research around MECC implementation should target more patient-facing service providers. Most documents submitted for the document analysis were outdated, therefore, not providing the current up-to-date picture of how MECC is being implemented throughout NENC. The present research was undertaken between March 2022 and December 2022, after MECC implementation was delayed/paused in many organisations due to COVID-19. Some of our findings reflecting the impact of COVID-19 on implementation may be too early to gauge an idea of the long-term impacts of COVID-19.

### Conclusions

We drew on the TDF and NPT in a process evaluation to understand the implementation of MECC. In particular, our findings suggest that the implementation process can be supported by a top-down approach, whereby organisational culture encourages MECC to achieve wider organisational goals. Recording of MECC was also highlighted as critical and potentially achieved through enhanced systems for recording MECC delivery. Availability of resources and support, and networking for shared knowledge could also facilitate the implementation process. Provision of training and resources with sufficient flexibility to accommodate local contextual and cultural adaptations is also essential, in addition to fostering implementation practitioner competencies and skills. Training should include solutions to overcome the barriers to cascading training, including adequate time and support to prioritise training.

## supplementary material

10.1136/bmjopen-2024-084208 online supplemental file 1

10.1136/bmjopen-2024-084208 online supplemental file 2

10.1136/bmjopen-2024-084208 online supplemental file 3

10.1136/bmjopen-2024-084208 online supplemental file 4

10.1136/bmjopen-2024-084208 online supplemental file 5

10.1136/bmjopen-2024-084208 online supplemental file 6

10.1136/bmjopen-2024-084208 online supplemental file 7

10.1136/bmjopen-2024-084208 online supplemental file 8

10.1136/bmjopen-2024-084208 online supplemental file 9

## Data Availability

Data are available on reasonable request.
