# Supplementary material for: Mapping regional implementation of ‘Making Every Contact Count’: mixed-methods evaluation of implementation stage, strategies, barriers and facilitators of implementation
Source: BMJ Open. 2024 Jul 22;14(7):e084208. doi: 10.1136/bmjopen-2024-084208 (PMC11268057; doi:10.1136/bmjopen-2024-084208)
Supplement: online supplemental file 1 [file bmjopen-14-7-s001.pdf]

Supplementary Material 1: Coding for the documentary analysis. Code: ✓= Included, X = Not reported

MECC Intervention Specification	Organisation 1	Organisation 2	Organisation 3	Organisation 4	Organisation 5
Areas Targeted					
Weight Management	X	✓	X	X	X
Physical Activity	✓	✓	X	X	X
Diet	X	✓	X	X	X
Smoking Cessation	X	✓	✓	X	X
Mental health and wellbeing	X	✓	X	X	X
Providers					
Primary healthcare professionals/ NHS specialists	✓	X	✓	✓	X
Health educators	X	✓	X	X	✓
Setting					
Range of venues in the community	✓	✓	✓	✓	✓
Format/Delivery					
Group	X	✓	X	X	X
Face-to-face	X	✓	✓	X	✓
Individual	✓	✓	✓	X	✓
Digital (supporting component)	X	✓	X	X	✓
Stage of implementation					
Planning	✓	✓	✓	✓	✓
Education and Training	✓	✓	✓	X	✓
Delivery	✓	✓	X	X	✓
Evaluation	X	✓	X	X	✓
ERIC Taxonomy					
Number of strategies	30	42	18	30	11

Normalisation Processing Theory (NPT) & Realist Evaluation (RE) framework					
**Context**					
Strategic intentions	✓	✓	✓	✓	✓
Adaptive execution	X	✓	✓	✓	✓
Negotiating capacity	✓	X	✓	✓	✓
Reframing organisational logics	✓	✓	✓	✓	✓
**Mechanisms**					
Coherence building	✓	✓	✓	✓	✓
Cognitive participation	✓	✓	✓	✓	✓
Collective action	✓	✓	✓	✓	✓
Reflexive monitoring	✓	✓	✓	✓	✓
**Outcomes**					
Sustainment	✓	✓	X	✓	✓
Normative Restructuring	X	X	X	X	✓
Relational restructuring	X	X	X	X	X
Intervention performance	✓	✓	✓	X	✓
Training					
Number of training quality markers	5	10	4	3	9
Pre-training evaluation	X	✓	✓	✓	✓
Post-training evaluation	X	✓	✓	✓	✓
