# Supplementary material for: Mapping regional implementation of ‘Making Every Contact Count’: mixed-methods evaluation of implementation stage, strategies, barriers and facilitators of implementation
Source: BMJ Open. 2024 Jul 22;14(7):e084208. doi: 10.1136/bmjopen-2024-084208 (PMC11268057; doi:10.1136/bmjopen-2024-084208)
Supplement: online supplemental file 2 [file bmjopen-14-7-s002.pdf]

Supplementary Material 2: Results of MECC mapping survey. Abbreviations: PA = physical activity, MHFA = mental health first aider, LA = local authority.

**Survey item**	Responses as defined for each question						
Organisation type (n = 34)	VSCE<br><br>4	LA<br><br>13	Healthcare<br><br>12	Government agency<br>1	Education<br><br>1	Public health (collaboration)<br><br>1	Other (not defined)<br>2
Length of time implementing/ delivering MECC in current role (n = 33)	Less than 1 month<br>6	1-5 months<br><br>4	6-11 months<br><br>7	1-5 years<br><br>14	6-10 years<br><br>2	11+ years<br><br>0	
MECC currently being implemented (n = 32)	Yes<br>29	No<br>3					
When MECC implementation began (n = 25)	Range = 2012-2022	Median = 2019					
Organisation's role in MECC implementation (n = 32)	Training<br><br>3	Delivery<br><br>3	Training & delivery<br>19	Other<br><br>7			
Stage of MECC Implementation (n = 27)	Planning<br>5	Education<br>3	Training<br>4	Delivery<br>6	Evaluation<br>2	Other<br>7	
Number of MECC conversations in preceding week (n = 12)	Range = 0-100	Mean = 22.12	Other:<br>N/A = 3<br>Countless = 1				
Proportion of service users seen within the preceding week that received MECC conversations (n = 11)	SD = 39.20	Mean % = 50.00					
Proportion of service users seen in the preceding week believe benefited from MECC conversations (n = 9)	SD = 37.81	Mean % = 67.77					
Staff groups involved/previously involved with MECC	HCPs<br><br>8	Social care<br><br>5	LA/council<br><br>10	VSCE<br><br>3	Community staff<br>5	Local businesses/ residents<br>3	Internal (e.g admin, HR)<br>7

implementation (n = 21)							
Staff groups plan to reach in future (n = 22)	HCPs<br><br>7	Social care<br><br>3	LA/council<br><br>11	VSCE<br><br>2	Community staff<br><br>3	Local businesses/ residents<br>2	Internal (e.g admin, HR)<br>7
Clients/service users delivered to (n = 18)	Patients/ service users<br>6	External partners<br>4	Internal staff<br><br>9	Social prescribers<br>2	Health advocates/ champions<br><br>1	N/A<br><br>3	
Clients/service users planned to reach in future (n = 15)	Patients/ service users<br><br>3	External partners<br><br>6	Internal staff<br><br>8	Social prescribers<br><br>1	Health advocates/ champions<br>2		
MECC infrastructures heard of/used (n = 21)	MECC directory website<br><br>10	MECC Organisational Implementation plan<br><br>11	MECC gateway website<br><br>21		MECC logic model<br><br>10	ICS training app<br><br>11	MECC quality marker for training guide<br>11
Organisations worked with to support MECC implementation/ delivery (n = 22)	VSCE<br><br>13	LA<br><br>16	Private sector organisations<br>8	Other<br><br>7			
Worked with other organisations (n = 22)	Yes<br>11	No<br>11					
Type of interactions with other organisations (n = 11)	Information exchanges<br>11	Resource sharing<br>10	Training<br><br>7	Other<br><br>0			
Further support from other organisations needed (n = 16)	None<br><br>7	Share best practice<br><br>3	Tailoring of resources<br><br>2	From external partners<br>2	With measuring<br><br>1	Identifying MECC leads<br><br>1	N/A = 1<br>Unsure = 1
MECC training received in last 18 months (n = 22)	Yes<br><br>16	No<br><br>4	No, but due to receive training<br>2				
When training received (n = 16)	Within last month<br>6	Within last 3 months<br>2	Within last 6 months<br>3	Within last 12 months<br>2	Within last 18 months<br>3		
Courses taken (n = 16)	Introduction to MECC<br><br>14	5 ways to wellbeing<br><br>4	Motivational interviewing skills<br>2	Behaviour change (all variations)<br>4	Specific topics:<br>MHFA = 0<br>Smoking = 2	Connect 5 (all levels)<br><br>10	Other<br><br>3

					Weight & PA = 6<br>Alcohol = 6		
Training sessions attended after invitation (n = 15)	All<br>10	Some<br>3	One<br>1	None<br>1			
Reasons for non-attendance to training (n = 6)	Someone else went<br>2	Competing priorities<br>2	Annual leave<br>1	Diary clash<br>1			
Training method (n = 20)	Face to face<br>14	Online<br>14	Self-directed online<br>3	Other<br>5			
Knowledge of number of training sessions received in organisation (n = 18)	Range = 0-52	Median = 2	Unknown = 5				
Duration of each training session (hours) (n = 18)	Range = 0-3	Mean = 2	Unknown = 1				
Health-related topics discussed with clients in last 6 months (n = 18)	Nutrition/ weight (combined score)<br>21	Physical activity/ exercise<br>12	Alcohol<br>10	Mental health and wellbeing<br>15	Smoking<br>11	Other: Sexual health = 6<br>Drugs/ substance abuse = 11<br>Other = 7	Prefer not to say:<br>1
Topics change from initial implementation (n = 22)	Yes<br>14	No<br>8					
How changed (n = 9)	Availability of tailoring<br>2	Expanded topics<br>2	Added Connect 5<br>1	Implementation focused<br>1	Confidence /awareness<br>3		
Type of MECC contacts with service users (n = 14)	1 to 1 face to face<br>11	Group face to face<br>5	1 to 1 telephone<br>8	Group online<br>6	1 to 1 online<br>5	Other<br>4	
No. of staff involved in MECC implementation/delivery (n = 22)	**0-10**<br>12	**11-20**<br>4	**21-50**<br>2	**51-100**<br>1	**101-200**<br>1	**200 +**<br>0	Unsure/ externally commissioned<br>2
No. of service users involved with MECC implementation/delivery (n =22)	7	2	1	5	0	3	Unsure<br>4

No. of service users delivered to (n =18)	6	2	0	3	0	5	Unsure 2
Impact of COVID on implementation/delivery	MECC service paused	Staff capacity issues	MECC training delivery	MECC contact with service users	Other	No change	
During lockdown (n = 20)	6	7	10	8	3	1	
Post lockdown (n = 20)	5	7	10	5	1	6	
