# Supplementary material for: Mapping regional implementation of ‘Making Every Contact Count’: mixed-methods evaluation of implementation stage, strategies, barriers and facilitators of implementation
Source: BMJ Open. 2024 Jul 22;14(7):e084208. doi: 10.1136/bmjopen-2024-084208 (PMC11268057; doi:10.1136/bmjopen-2024-084208)
Supplement: online supplemental file 3 [file bmjopen-14-7-s003.pdf]

Supplememtary Material 3: interview topic guide with TDF and NPT coding.

Mapping 'Making Every Contact Count' (MECC) implementation across the North East and Cumbria: A comparative analysis of delivery models, service reach and system-level relationships

All participants should have received a copy of the information form and consent form prior to the discussion. Ask all participants to confirm that they have received both and have electronically returned a signed consent form. Ask participants to also confirm on the recording that they are happy to continue with the discussion. Continue to read the paragraph below and allow for people to ask any questions.
Introduction [please read this to the interviewee before the interview takes place]

My name is Caroline Charlton/Angela Rodrigues, and I am interviewing you on behalf of a research project being conducted by Northumbria University. Knowing your experiences regarding MECC implementation and delivery with NENC is very important because it means we can gain an accurate picture of what is currently happening and develop recommendations towards future practice.

If you don't mind, the discussion will be recorded and I will take a few notes. The notes and the recording will be kept completely private, meaning no names or identifiable information that you mention in the recording will be used when it is typed up, meaning we will not use your name or any other information that could be used to identify you, having said that, you may want to stick to first names and avoid using identifiable information for the sake of the recording, but this is entirely your choice. Are you happy to go ahead with the discussion?

About the stakeholder
Can you tell me about your job role and why you got involved in the implementation of the MECC programme? (Prompt: Did you clearly understand your role within the implementation of the MECC programme? How were you informed of the MECC programme?)

1. First of all, can you tell me a little bit about:
a. Your role in [name of organisation/service]?
b. Your involvement with MECC?
c. What do you do as an implementer/deliverer of the MECC programme? (Social professional role and identity)

Early Implementation (for everyone)

2. What does MECC mean to you?

3. How important do you think the MECC programme is in general? (emotion)

4. How aware were you/ your organisation about the MECC intervention before it was implemented within your organisation? (Knowledge; NPT coherence)
a. What kind of conversations were you/ are you having in your organisation regarding MECC? (Implementation outcome: penetration)

5. What knowledge do you need/did you need to know in order to implement the MECC programme? (knowledge)
a. What kind of information or evidence were you aware of that showed whether or not the intervention would work in your setting? (knowledge; implementation outcomes: appropriateness)

6. Why was the MECC programme implemented/why interested in implementing the MECC programme within your setting, what motivates you? What did you hope to achieve? (goals) (local, state, or national performance measures, policies, regulations, or guidelines influenced the decision to implement the intervention, organisational goals? health, community, making a difference;) (Memory attention and decision making; intentions; implementation outcomes: adoption; NPT coherence)

a. financial or other incentives influenced decision? Advantage for your organization compared to other organizations in your area? (Memory, attention, and decision making; Reinforcement)
b. Acceptability of MECC (satisfied with the content, complexity, comfort, delivery, credibility) (intervention outcomes: acceptability)
c. Who is/was responsible for the decision (or what was/will the decision process) to implement MECC within your organisation? (Decision from the top? If so, why?) (Memory, attention and decision making; social professional role and identity; Implementation outcome: penetration)
d. Reactions to the suggestion of implementing MECC in your organisation? conflicting beliefs about the implementation of the MECC programme within your organisation? (Colleagues, staff, etc) (social influences)

7. How did/do you feel about getting involved initially?
a. Expectations (beliefs about consequences)
b. fit with organisational ethos/ your professional role /identity? /Existing work processes and practices (professional role and identity; implementation outcomes: feasibility; NPT cognitive participation) Steps taken to integrate into your organisation? I.e., was a pilot ran?

8. What kind of support or actions did you receive/are you receiving/hoping to receive from leaders (LA’s, government, others?) to help make implementation successful? (Social influence/support; Environmental context and resources)

If just starting/not yet started:

a. Do you know who to contact for support regarding MECC implementation/delivery? If so, who?

For all

b. To what extent do you network with colleagues or people in similar professions/positions outside your setting? i.e., MECC strategy group, other organisations implementing MECC, national entities. Does your organisation encourage you to network? What kind of information exchange do you have with others outside your setting, either related to the MECC programme, or more generally about your profession?

9. What staff, facilities and resources are/were needed in order to implement the MECC programme? (what is lacking?) (Environmental context and resources)

In your opinion, what do you think are the main important elements for successfully implementing the MECC programme?

8. How do other organisations and/or programmes in your area impact on your implementation of MECC? (Prompt: If any, are these helpful or not?)

How do you feel the implementation of MECC has gone? Prompt: is this how you might have expected? (why/why not/ in what ways?)

a. What kinds of information/resource and materials about the intervention were made available to you? How did you expect to procure necessary resources? Perception of quality of resources (materials, packaging, and bundling of the MECC programme for implementation?)
b. How did available materials/resources affect MECC implementation in your setting?
c. What MECC infrastructures have you heard of/ used (e.g., MECC gateway website, strategy group)

10. What costs were incurred in order to implement MECC into your organisation? (available funding to support?) (Implementation outcomes: implementation cost)

11. Did the MECC programme/will the MECC programme replace or compliment other programmes or processes within your organisation?

   a. If yes, Are there any benefits of the MECC programme over similar/other programmes used within your organisation? (knowledge)

   b. Are there any disadvantages of the MECC programme over similar/other programmes used within your organisation? (knowledge)

   c. How does MECC compare to other similar existing programs in your setting?

Ongoing roll-out (questions not relevant for those just starting/not yet started)

12. What does MECC look like in your organisation now? (NPT collective action)(How has funding been used? Any change from initial plans? Tailored to own organisation? If so, how?

I.e., infrastructure of your organisation (social architecture, age, maturity, size, or physical layout))
a. How have staff responded to MECC training? (what is the training structure in your setting? [skills], confidence in the skills and capabilities of staff to deliver MECC programme (more confident is some areas then others?) [skills; beliefs about capabilities], is there any further training needed? (refresher/different training needed?) [skills] Could own skills within MECC be improved? [skills; NPT cognitive participation]
b. Have there been any changes to organisational processes due to MECC (e.g. staff inductions, job descriptions, organisational policies, MECC champion, COVID-19)?
c. **Ask all participants including those just starting/not yet started**: How is information about MECC recorded (e.g. interventions, onward referrals)? (Challenges? Any changes or development to recording systems? Do you have a system in which you use to record/monitor MECC implementation/delivery? How do you know the MECC programme is being delivered as intended? (behavioural regulation; implementation outcomes: fidelity) optimistic about successful implementation of MECC? (optimism)
Lack of documentation, why? Use of logic models etc? For those just starting/not yet started: Do you have an implementation plan?

13. **Ask all participants including those just starting/not yet started**: What do you think the impact (unintended or unexpected consequences) or benefits of MECC have been/will be: (beliefs about consequences; NPT reflexive monitoring)
a. On frontline staff's role and practice: Do you engage in practice consistent with policy recommendations? (COM-B**)). If delivering MECC**: How do you decide when to use MECC conversations? Is there specific criteria or guidance you follow? (Memory, attention and decision making) What impacts positively/negatively on opportunities to deliver MECC interventions in your workplace?
b. On the organisation
c. On clients (How do you know if there has been any impact? Do you receive feedback? If so, what feedback have you received? (reinforcement); Were service users needs/preferences considered when deciding to implement MECC? Staff awareness of service users' needs and preferences? What are your beliefs on patient motivation? (COM-B) Fear of offending the patient (COM-B)
d. On staff's own health and wellbeing behaviours (including your own)
e. On wider networks or other organisations you work with?

14. Are there ways MECC could be modified or improved to better suit your organisation? (Would you do anything differently if involved with MECC again?) (reinforcement)
a. Who decided (or what is the process for deciding) whether changes were needed to the MECC programme so that it worked well in your setting? (Memory, attention and decision making; social professional role and identity)

15. How well do you think the intervention meets the needs of the individuals served by your organisation? (optimism)
a. improving the performance of the MECC programme holds the potential to improve service user care in the future? (optimism)

16. Do you plan to continue MECC within the organisation? (If so, how?) (intentions)

a. Maintenance of MECC in organisation (Implementation outcomes: sustainability)

17. How rewarding is implementing/delivering the MECC programme? (reinforcement)

Prompts: How might the implementation of MECC be improved?
How sustainable do you think the MECC programme is in the long term?

Is there anything that you would like to mention or add that you do not think we have covered?
