# Supplementary material for: Mapping regional implementation of ‘Making Every Contact Count’: mixed-methods evaluation of implementation stage, strategies, barriers and facilitators of implementation
Source: BMJ Open. 2024 Jul 22;14(7):e084208. doi: 10.1136/bmjopen-2024-084208 (PMC11268057; doi:10.1136/bmjopen-2024-084208)
Supplement: online supplemental file 4 [file bmjopen-14-7-s004.pdf]

Supplementary Material 4: Mapping survey results. Note, survey items have been compiled for disagree and agree (e.g 'Disagee' to include 'strongly disagree').

**Survey item**	**Mean**	**SD**		
**COM-B**				
Do you believe that MECC interventions are effective in helping patients make lifestyle changes?<br>0-100% (n = 19)	(%) 76.68	15.12		
How would you rate your awareness of the MECC policy?<br>0-100% (n = 19)	(%) 83.36	16.76		
How much time is spent on implementing/delivering MECC interventions in patients who may benefit?<br>0-100% (n = 16)	(%) 47.18	31.07		
**NoMAD**				
MECC normal part of role 0-100%<br>(17)	(%) 71.64	32.31		
**Survey item**	**Agree (%)**	**Neither agree nor disagree/ uncertain (%)**	**Disagree (%)**	**Other (specified) (%)**
**COM-B**				
Do you believe that you have sufficient knowledge and awareness of the appropriate effective behaviour change techniques to confidently implement/deliver MECC? (n = 20)	95	5	0	-
Do you believe that you have had the appropriate training and skills to implement/deliver MECC? (n = 19)	94.7	5.3	0	-
Do you feel able to select and use brief lifestyle behaviour change techniques that help individuals take action about their lifestyle choices? (n = 19)	100	0	0	-
Do you believe that you have the appropriate resources and equipment to be able to implement/deliver MECC? (n = 20)	95	5	0	-
Do you feel that you have support from colleagues to implement/deliver MECC? (n = 21)	95.2	4.8	0	-
**NoMAD**				
C1: Staff have a shared understanding of the purpose of MECC (n = 19)	68.4	10.5	10.6	10.5 (not relevant)

C1: I can see the potential value of MECC for my work (n = 20)	100	0	0	-
C2: There are key people who drive MECC forward and get others involved (20)	100	0	0	-
C2: I believe that participating in MECC is a legitimate part of my role (n = 20)	95	0	0	5 (not relevant to my role)
C3: I can easily integrate MECC into my existing work (n = 19)	94.7	0	0	5.3 (not relevant at this stage)
C3: Management adequately support MECC (n = 20)	85	15	0	-
C4: The staff agree that MECC is worthwhile (n = 20)	75	20	0	5 (not relevant at this stage)
C4: I value the effects MECC has had on my work (n = 19)	89.5	5.3	0	5.3 (not relevant to my role)
