# Supplementary material for: Mapping regional implementation of ‘Making Every Contact Count’: mixed-methods evaluation of implementation stage, strategies, barriers and facilitators of implementation
Source: BMJ Open. 2024 Jul 22;14(7):e084208. doi: 10.1136/bmjopen-2024-084208 (PMC11268057; doi:10.1136/bmjopen-2024-084208)
Supplement: online supplemental file 5 [file bmjopen-14-7-s005.docx]

Supplementary Material 5: Themes, sub-themes and example codes categorised by TDF/NPT domains with examples of corresponding quotes. Quotes are labelled according to the workshop number they were extracted from (workshop 1 (W1), 2 (W2) or 3 (W2)), or participant interview number (e.g P12).

| **NPT;TDF** | **Themes** | **Barrier/ Facilitator/Mix** | **Sub-themes** | **Example codes** | **Corresponding quotes** |
| --- | --- | --- | --- | --- | --- |
| Coherence;  Knowledge | Variation in definition/ meaning of MECC | Mixed | MECC is an underlying approach/underpinning foundation, not an intervention  MECC offers a consistent approach to optimise conversations | MECC as an underpinning approach rather than an intervention  MECC provides consistent a strategy for behaviour change  MECC increases accessibility of health promotion and prevention  MECC as a patient centred approach  MECC as opportunistic approach to discuss health and wellbeing | W1: *‘Underpins our work with communities’*  W2: *‘An approach to behaviour change utilising daily opportunities and interactions to influence health and wellbeing’*  W1 (padlet): *‘ensuring a consistent systematic*  *approach'*  P10: *‘it’s an alternative way that makes health improvement everybody’s business’*  *P4: ‘it puts the person right at the centre.. it’s kind of opening up options and providing those pathways’*  P6: *‘just got to wait for them to have an opportunistic conversation’* |
|  | Knowledge/ awareness of MECC |  | Existing awareness of MECC, either personally or through professional roles  Began MECC role with no existing awareness of MECC | Personal prior knowledge of MECC, particularly for those from health backgrounds  MECC concepts already part of professional role and identity  Limited awareness/knowledge of MECC within organisation before implementation (mixed) | P13: *‘general practice and primary care are more aware of the concepts than others’*  *P6: ‘I’d had a role previously that was MECC orientated’*  *P4: ‘I didn’t know a lot about it before I started’* |
|  | Knowledge needed to implement/ deliver MECC |  | Knowledge required of MECC itself  Knowledge required of the organisation | Knowledge of available resources  Knowledge of what MECC is (including theory and delivery)  Knowledge of up-to-date information  Knowledge of organisational fit/ implementation process needed  Knowledge of other organisations/ networks | P7: *They’ve got a much greater awareness of where they can find resources now which was maybes putting them off having a conversation in the first place’*  P17 *‘I think it’s very lendable and very acceptable once you actually break the barrier and explain what MECC is’*  P11: *‘about actually what’s changed in terms of guidance and recommendations’*  W1: *‘we also need to know where to signpost or send people which again has changed significantly over recent times’*  P1: *‘But what I needed to know was where were the opportunities within the organization to do that’*  P10: ‘*I don’t know all the organisations in (name of location) and what they deliver’* |
|  | Evidence (or lack of) | Mixed | Lack of existing evidence of outcomes for service users  Use of evidence to facilitate MECC implementation  Low value placed on attaining evidence | Lack of evidence to draw upon to ‘sell’ MECC  Issues surrounding evidence base strength  Pilots ran to ascertain organisational suitability/fit  MECC conversations easier to measure than outcomes  Awareness of evidence  Implementation should happen anyway | P9: *‘maybe we need a bit more resources and toolkits and data to just back up the argument as to why it’s important’*  P4: ‘*what I’d love to see actually is a little bit stronger evidence and case studies to go along with that’*  P10: ‘*at the minute we’re kind of in the pilot to try different things, see what works, see how we can work with different people’*  P5: *‘we’ve got some really good data around how many conversations have happened’*  P9: *‘it comes back to the national evidence base of kind of the benefits of MECC and brief intervention training’*  P3: *‘So, I think what we have to do is take a leap of faith erm on the understanding that erm behaviour change around the prevention agenda is important and it will help. Erm you know, but that we’re not necessarily going to be able to define how important it is’* |
| Cognitive participation; Social/ professional role and identity | Roles, responsibilities, and accountability | Mixed | Facilitator of clearly identifying MECC roles  Challenges of adding MECC to existing roles | Named contact required both regionally and within organisation  Belief that MECC is an additional responsibility to add to increasing workload  MECC viewed as ‘not my job’ | P16: *‘having someone who leads on it, is the go to person, has been really important’*  P10: *‘it’s just hard to get people to understand that, isn’t it, that actually we could all just do this. It doesn’t need to be a burden’*  P15: *‘I think it’s challenging to roll out because people quite often are reluctant, not my job’* |
|  | Feeling of involvement with MECC | Facilitator | Personal feeling of involvement with MECC that extends outside of role (facilitator) | Enthusiastic individual (motivated)  Fundamental belief in MECC (helps to promote MECC outside of roles and responsibilities) | P5: *‘if you’re passionate about something and you’re capable of delivering a message it really helps’*  P6: *‘I always like want to put MECC where I am no matter what role that I’m in’*  P8 *‘I’m MECC chipped now, so I talk about it all of the time whenever I’m doing anything. …once you’re chipped you’re chipped’* |
| Cognitive participation/ collective action; beliefs about capabilities | Process to increase staff confidence to have meaningful conversations | Mixed | Confidence in initiating and holding MECC conversations | Belief that MECC increases confidence (facilitator)  Lack of confidence results in missed opportunities (barrier) | P13: *‘it’s a framework for people to use’*  P11: *‘I think MECC gives people that permission’*  *P7: ‘as opposed to staff panicking that they’ve got to have a MECC conversation because they don’t know what to say’* |
|  | Difficulty of raising certain issues with service users | Mixed | Belief that this difficulty can be reduced/avoided (facilitator)  Fears prevent approaching MECC conversations (barrier) | Belief that MECC provides guidance on broaching the conversation sensitively  Fear of offending  Specific topics particularly difficult to approach (most commonly weight) | P7: *‘it does come back to how you start that conversation and not doing it as a finger wagging’*  P6: ‘*I think if that if MECC’s done right it shouldn’t ever offend somebody’*  P10 *‘I think there’s a massive thing about being afraid to overstep the mark’*  P7: *‘I think it’s more so weight seems to be the one where staff have the most difficulty’* |
| Cognitive participation; behavioural regulation | Maintenance of MECC in organisation | Facilitator | Embedment of MECC within organisational processes  Maintaining staff buy-in | Some inclusion within staff induction, appraisals, job descriptions, and return to work interviews  Incorporating MECC into all work streams increases sustainability  Senior leadership buy-in essential to maintain promotion of MECC  Named coordinators/leads to help maintain buy-in | P1: *‘we changed like some of the human resources staff to when they were doing like return to work interviews or things like that’*  P3: *‘we’re linking in with all the other workstreams which just like makes it a bit more sustainable’*  P18: *‘you need senior leadership buy-in’*  P15*: it’s how we as in myself and the strategic lead have an impact on helping people understand the importance of MECC’* |
|  | Recording/ monitoring MECC | Mixed | Challenges of recording/measuring MECC  Adaptations to enable recording/measuring of MECC | Difficult to record outcomes of MECC    Difficult to attribute effectiveness of MECC  Use of existing systems to record MECC conversations | P10: *‘I think that is something actually that we lack.. how do we measure the success of MECC because it’s really hard. Because it’s brief intervention, they’re gone, and you might not see that person again’*  W3: *‘really difficult even without variability across staff groups’*  *P9: ‘it’s obviously just really difficult to demonstrate that direct causality’*  W1: *‘Biggest challenge is demonstrating outcomes and causality in relation to behaviour change as a result of MECC interventions’*  P1: *‘So we knew for every admission if they'd had this smoking and alcohol status recorded and.. depending on the outcome, if it had a referral or not. ..but that's embedded. It's in our electronic records now’* |
|  | Evaluation | Mixed | Collection of formal feedback (e.g pre-post surveys)  Qualitative feedback (informal) | Commonly used for training  Less commonly used for implementation  Used to refine training  Easier to gain during in-person training  Qualitative feedback reduces amin time of trainee and recipient | P15: *‘we tend to do evaluations after every course’*  P13*: I don’t know where we’re at with it currently. I don’t think I could evaluate that easily’*  W1: *‘Challenges with gathering 6 month follow up evaluation to understand how MECC continues to be used in practice’*  P18: *‘I’ll review that feedback and see like maybe what changes we can implement’*  *P14: ‘I delivered one session online and that’s a nightmare because people don’t then send you the feedback’*  P14: *‘we find a lot of it informally through listening’* |
|  | Implementation process | Mixed | Embedment of MECC within organisational programmes and policies  Mostly top-down implementation approach  Stages of implementation | Aim to embed within organisational policies  MECC as a ‘golden thread’ to existing programmes  Top-down approach most common and valued  Less common: Bottom-up approach (e.g raising awareness, driven by one invested person, and culture change)  Cycle of evaluation and refreshment of MECC  Pathways - regarding strategic implementation  Lack of use of documents for implementation (action plan/logic model) | P12: *‘it’s going to be embedded in our staff health and wellbeing policy so that gives us that leverage’*  *P14: ‘so MECC is even more embedded and not running in parallel which I’d rather it didn’t. I’d rather it was embedded totally’*  W2: *‘It helps to have Chief Exec onboard and built into Council strategies’*  P16 *‘it’s always resisted by staff when you tell them oh this is another thing that nationally you have to do.. so it has to come from the.. ground up’*  P1: *‘so it was implemented because I managed to get the board to sign up to a public health strategy’*  P11*: ‘actually in that process of refreshing now’*  P1: *‘We did change some of the clinical pathways. So some of the electronic pathways we embedded it in.’*  P9: *‘I think we probably did initially and then it probably just went by the wayside over time’* |
| Collective action; social influence | Networking with other organisations | Facilitator | Sharing of knowledge and resources  Supportive networking environment  Supporting MECC implementation | Knowledge exchange (e.g resources, information exchanges)  Shared learning  Facilitated by MECC steering/strategy group with MECC coordinator as central  Healthcare and LA/VSCE mainly communicate in silos  Partnerships with organisations provides new pathways for implementation | P2: *‘but actually we’re not just working with them, they’re working with us.’*  W2: *‘At first it seemed to be in different sources but as I became more confident and connected I felt better able to access what I needed’*  P14: *‘That sharing of practice. And having that mix of staff who have been involved for a long time who can share their knowledge and experience’*  P15: *‘[MECC coordinator] has been a complete lifeline’*  W1 (padlet): *‘Informing and informed by regional group, national policy, and local networks’*  P7 *‘you just get on and implement your own and I think part of that is because I still think the approach is very different in a trust to what it is in a workplace or a local authority area’*  P10: *‘what I’d want from them is a key person or two who will attend our training to then disseminate it to their workforces’* |
|  | Networking within organisations | Facilitator | Healthcare more likely to communicate within organisation than between | Building internal networks for collaboration  Support internally | P16: *‘it becomes more part of a professional network, so for example nurses, different nurses, different staff networks can take that forward’*  P8: *‘we have a weekly MECC meeting’* |
| Collective action/ cognitive participation; environmental context and resources | Materials/ resources |  | Available resources mainly viewed positively  Lack of resources to implement MECC (barrier)  Tailoring of resources | Improvement in resources  Usefulness of resources  Lack of action plan /logic model, slows implementation during staff handovers  Tailoring to the organisation/location  Tailoring to the setting  To ‘improve’ existing resources | P11: *‘fast forward post COVID and now I think the resources are much easier to use’*  *P1: ‘all of that was all really, really helpful’*  P13 *‘key personnel have changed so actually then they take time to embed into the system again don’t they?’*  P12: *‘we will be developing that localism around slides, leaflets, web access ourselves’*  P17: *‘because you adapt them slightly to your setting, I had my own slides’*  *P10: ‘it’s probably a little bit different’* |
|  | Costs/ funding to implement/ deliver |  | Availability of funding (mixed)  Low funding not a barrier | Funding mostly only for staff capacity  Mostly, the only physical costs are resources, materials, and venues for training  MECC as low cost to implement | P13: *‘I haven’t been made aware of any funding’*  P14: *‘low cost really, printing costs and things like that’*  W1: *‘Low cost at-scale approach’*  P10: *‘it should take a low resource as well to have an impact’* |
|  | Staff capacity | Barrier | Staff capacity issues to implement MECC  Staff capacity issues to deliver MECC conversations | As a barrier to training for those who would ‘benefit from it the most’  Barrier of competing priorities  MECC conversation takes longer than initially expected | P10: *‘one of the main things is the capacity.. We haven’t got time to put somebody on the training even though it’s short’*  P11: *‘I have, like lots of people, have lots of different parts of my portfolio’*  P1: *‘You’ve started a discussion and for all it’s meant to be a brief intervention, the client then wants to have a further discussion’*    W1 (padlet): *‘Don't have capacity to roll out delivery as yet’* |
|  | Mixed influence of COVID | Mixed | Positive effects of COVID on MECC implementation (facilitator)  Challenges of COVID for MECC implementation (barrier) | COVID encouraged movement to online training  Improved accessibility of training and resources  Improved dissemination of training through movement to online  Acceptability of online training model  Reduced staff capacity for MECC (due to COVID) | W1: *‘COVID has been challenging, but it has provided an opportunity to use different methods of delivery’*  P6: *‘there was no priority given to any geography. Everybody was on the same footing. Everybody joined the Teams meeting like everyone else’*  P8: *‘I was able to deliver it to 200 staff in one go because I was doing it virtually’*  W1: *‘As a result of Covid we've been able to train more of our workforce in MECC to support our response’*  P6: *‘a lot more people wanted virtual training because they weren’t having a day out of the office’*  P13: *‘everything paused’*  W2 *‘MECC was definitely not prioritised during the pandemic and lost momentum as a programme’* |
|  | Organisational fit | Mixed | MECC fits within organisational practices  Mixed acceptability of MECC | Compliments existing programmes  Fits with organisational policies and principles  Initial apprehension around organisational fit  Acceptance of MECC- already practice it | P3: *‘I think it’s complementing all the work that’s been done’*  P16: *‘it definitely fits with the organisation and it’s values and the strategy’*  P1: *‘So there was a real mix or it was just like this won't work for these people’*  P11*: ‘they do a lot of MECC but they don’t call it MECC’* |
| Cognitive participation/collective action; skills | Training |  | Cascade model of training (mixed)  Ability to tailor training valued but not always feasible (Mixed)  Embedding into existing training  Training increases confidence and competence | Train the trainer model favoured for implementation    Cascade model stops at train the trainer  Support needed for cascading- low confidence in delivering training  Refresher training needed  Use of national guidlines to develop tailored training  Tailoring to organisation, setting, or location  Not always possible depending the training group  Barrier to MECC training delivery  Increased confidence  Increased competence | P7: *‘we can just do it at a much quicker speed with the train the trainers’*  W2: *‘the gold standard for us will be the train the trainer model’*  W1: *‘Many end up trained, few end up utilizing.’*  P11: *‘some staff had been asked to do a train the trainer but then didn’t have the confidence or the skills to deliver’*  W1: *‘I attended a Train the Trainer session online but it was only an hour long and didn't feel it was enough to then deliver training myself’*  P5: *‘refresher training would be helpful’*  W1: *‘I developed the original [city] MECC offer based on good practice nationally’*  W1 (padlet): *‘there are challenges, much of which is making MECC right for different teams/types of front facing engagement - some MECC conversations fit better than others depending on the organisation*  *and that's where you have to start’*  W1: *‘I think it has to be fluid after initial training to suit different environments and different trainers or communities’*  P9: *‘the trickiest bit is just getting that balance where you’ve got multiple people from multiple backgrounds’*  P17: *‘an organisation isn’t going to take on additional training if they can embed it into what they already do’*  P1: *‘people’s level of confidence seem better’*  W1: *‘Competence and confidence to know how to use the 3A’s model’*  P6: *‘the training was the major thing to be able to have them conversations’* |
| Cognitive participation; reinforcement | MECC rewarding to implement/ deliver | Facilitator | Positive experience of MECC implementation and delivery overall | Rewarding when able to see progress  Implementing MECC is a challenge but beneficial | P11: *‘I really enjoy the programme. I’ve found it very rewarding to be part of it’*  P15: *‘it is very rewarding for me as an employee but actually getting to that point has been challenging’* |
| Reflexive monitoring; beliefs about consequences | Impact of MECC on staff | Facilitator | Improved health and wellbeing outcomes for staff | Improved health and wellbeing  Improved awareness of their own health and wellbeing | P12: *‘some of this is around a happier, healthier workforce’*  P17: *‘raising awareness of MECC is also about raising awareness of your own health and wellbeing’* |
|  | Impact of MECC on the organisation | Mixed | Favourable outcomes on organisation (facilitator)  Potential negative outcomes for organisation (barrier) | Improved image of organisation  Ability to provide care earlier in the care pathway  Risk of increased complaints | P14: *‘it’s about improving that service for people and having that positive feedback’*  P5: *‘there was an expectation that if we don’t do this, financially down the road.. we’re always going to be reacting to disease and not trying to stop it’*  P5: *‘the risk that we have identified in our risk log, we’re going to see an increase in complaints’* |
|  | Impact of MECC on clients/service users and the community | Mixed | Direct impact to patients (mixed)  Impact for wider scale change | Improved service and care  Empowered to self-care through increased knowledge  Difficult to see change (small impact, long period of time)  Culture change to prevention | P13: ‘*hopefully they’ll be getting better service because they’ll be getting better interviewing and techniques’*  P7: *So, you’re giving the patient the information to enable them to go and do something about it themselves’*  P10: *‘I don’t think anybody, if they have, it will be really minimal’*  P2: *‘I think it is one of the most important programmes that we have for prevention’* |
| Reflexive monitoring; goals | Goals for improving/ modifying MECC | Mixed | Goals to improve implementation process of MECC  Goals to improve measurability of outcomes of MECC | Make MECC general practice throughout through culture change  Embedding MECC at scale (creating dedicated pathways, training rollout, working with partners, embedment into policies and processes)  Goals for measurement of MECC  Development of case studies | P5: *‘the best outcome would be to ask anybody in the organisation what MECC is and they’d be able to tell you’*  *P9: the only way we could do that I suppose is getting that MECC culture embedded across the whole borough’*  P7: *‘where we are reviewing policies that are related to MECC where can we thread it into policies’*  W3: *‘to stop there being a massive drop out, we want to keep the number up by giving additional support’*  P12: *‘what we want to try and do, and we’re working on it at the moment, is how we can have a process to glean from people the impact it’s had on them or people around them’*  P12: *‘we want to try and build something in where at least we get some sort of feedback even if it’s more qualitative from team leaders’* |
|  | Goals that align with MECC implementation | Facilitator | MECC helps achieve public health goals to improve health and wellbeing | Helps to achieve organisational goals  MECC aligns with wider scale goals (e.g prevention agenda, person-centred care, reducing health inequalities)  MECC facilitates initiatives within the organisation (e.g Stoptober, Active Hospital) | P1: *‘We were awarded active hospital status.. and that was all around the concept of MECC’*  P7: *‘we’re trying to look at everything through like a health inequalities lens and I think again MECC is another means of doing that’*  W1 (padlet): *‘great for engagement and communication of public health issues’*  P5: *‘I guess that’s kind of MECC is part on delivering on active hospital’* |
