# Supplementary material for: Mapping regional implementation of ‘Making Every Contact Count’: mixed-methods evaluation of implementation stage, strategies, barriers and facilitators of implementation
Source: BMJ Open. 2024 Jul 22;14(7):e084208. doi: 10.1136/bmjopen-2024-084208 (PMC11268057; doi:10.1136/bmjopen-2024-084208)
Supplement: online supplemental file 6 [file bmjopen-14-7-s006.docx]

Supplementary Material 6: recommendations in accordance with the APEASE criteria, and as amended through discussion with stakeholders during workshop 3.

| **Initial recommendation** | **Feasibility of recommendation in accordance to APEASE criteria** | **Discussion during workshop 3 (in accordance with APEASE criteria)** | **Quotes from Workshop 3** | **New recommendation** | **Example delivery** |
| --- | --- | --- | --- | --- | --- |
| Implement further staff resources to combat staff capacity/time to deliver/implement issues (hiring people to specifically deal with MECC) | Low across all criteria, particularly affordability. Requires reconsidering | Implementation is driven by top-down processes  Increasing staff capacity may be costly and unrealistic Resource strategy, infrastructure and materials viewed as just as or more important than staff capacity to deliver MECC | *‘it’s needed, but who’s gonna pay is my big question’*  *‘Maybe about getting it to an organisational level where they’re supporting buy-in, mechanisms and infrastructure to sustain and develop MECC’* | Create a standardised infrastructure and strategy to combat delivery/implementation issues | Producing a ‘living’ logic model to improve efficiency of implementation, particularly for staff handovers  A hierarchical structure of MECC leads and MECC champions |
| Managing competing priorities: Implement organisational lead for MECC within organisation with sole responsibility to drive MECC implementation/delivery forward (admin support) ​ | Low, particularly around affordability and equity. Requires reconsidering | MECC lead can act as a pillar, but responsibility should be dispersed as MECC grows  Buy-in from senior management seen as essential | *‘who will get that role, and will they be given time to do that, or is it on top of what they’re already doing, and then you get the discussions around this is my responsibility, how can I do this?’* | Ensure buy-in from senior management to facilitate a change in organisational culture | Set up steering groups, ensure MECC is a standardised item on team meeting agendas, organise a credible source to talk about MECC at the organisation |
| Encourage further ‘buy in’ of MECC from additional pathways/organisational departments by disseminating evidence of effectiveness of the programme | High, relatively consistent across all criteria | Sharing of knowledge and experiences with other workforces was considered to be favourable | *‘There’s nothing wrong in our organisation with sharing that with other departments, and I think that’s probably a key part of the implementation of MECC’*  *‘I think absolutely it should be the way forward and it’s what we’re trying to do’*  *‘any buy-in can only be a positive, it’s just getting the buy-in’* | Remained the same | Sharing case studies that are relevant and applicable to trainees to demonstrate understanding of their role |
| Create supportive mechanism to put in place to support training and cascading after receiving MECC training. Potentially built into MECC regional offer | High, but particularly low in equity | Concerns around staff capacity and cost  Support system and expectations for the train the trainer model should be made explicit at advertising and sign-up stages | *‘people have attended the train the trainer training, and they’re a little bit unsure of expectations regionally’*  *‘when I get to train the trainer expectation slide, some people are shocked to learn they have to deliver’* | Create a support system and expectations around cascading and make these explicit at sign-up and recruitment stages of MECC cascading training | Offering a support package after cascade training e.g monthly peer support meetings and topic-specific sessions  Include cascade expectations (e.g 4 sessions over 12 months) on MECC cascade training flyer |
| Further develop/utilise online training/networking model to increase uptake in MECC training and networking opportunities (reduce inequalities i.e., travel requirements) ​  AND  Ensure core MECC resources are easily accessible and provide short e-learning on how to use these resources and how to tailor for organisational fit without losing the consistency of the MECC message. ​ | High, particularly for effectiveness and acceptability | Face to face training favoured to allow sharing of ideas and experiences, although online options considered essential for ensuring accessibility for those in a range of sectors and locations  Tailoring of training and resources help retain relevance | *‘I much prefer face to face, you’ve got my full attention’*  *‘I think it’s about having that variety’*  *‘Whilst MECC principles don’t change, it’s how you deliver that to be the champion of that really’* | MERGED: Allow for tailoring of training (hybrid model of online and face to face) and resources to attain organisational fit without losing the consistency of the MECC message. Provide short e-learning on how to use and tailor these resources | Core MECC training available in face to face, online, or self-paced formats, and core training resources available to amend according to the organisation, setting, and occupation |
| Implement MECC throughout organisational policies to ensure good practice and raise awareness of the MECC programme | Medium, particularly low for practicability | Acknowledged as important but will take time for MECC to become embedded into policies across the organisation | *‘We would probably have this as a longer-term aim, it is going to be a difficult one to implement, because there are so many trust policies that MECC could thread through’*  *‘changing culture is important, but it’s not an either or, so you need to change the policies also to change the culture’* | Remained the same, but acknowledged to be a long-term goal | MECC as part of staff inductions and return to work interviews |
| Create a standardised way to record/monitor MECC implementation/delivery across the region (to provide more structure) - how to embed within current systems – ensure everyone capturing the same data to improve measurability**​** | Medium, particularly low in practicability and equity | Recognised as more attainable for desk-based occupations  Organisations have their own recording systems and specific goals for outcomes of MECC | *‘Great recommendation, but often very difficult to do because of the different systems used by different organisations’*  *‘Some core things would be great, but then you do need to tweak them to your organisation’* | Create a consensus of what data to collect around MECC implementation and delivery across the region and embed within current systems, with allowance for organisational tailoring | Agree on core items to measure during regional MECC strategy group  Adding a ‘delivered MECC’ tab to existing internal recording systems |
| Incorporate the use/further of PPI to ensure clients/service users are being delivered MECC as intended – collect qual feedback to provide evidence of implementation effectiveness (i.e., case studies) | Medium, particularly low for side effects and equity | Worries around the outcome of PPI being divergent to MECC plans  PPI and the creation of case studies should be separated  Question of feasibility of capturing case studies, although important to attain | *‘I think in essence it's really good, I think we should be telling stories and using patients to be able to do that’*  *‘They might not recognise that discreet MECC conversation 3 months ago, was the reason they changed their behaviour later down the line’* | Consider how organistional and setting specific case studies could be developed to provide evidence of optimisation of implementation | Drawing upon setting and organisational specific case studies to both present to senior management and utilise for training |
| N/A (newly added) | N/A | Evidence of effectiveness of MECCC on service user outcomes considered important to encourage buy-in from senior management | *‘keen to know it is worth it, given that we have put lots of training in’*  *‘How do we evidence them, because we haven’t worked that out yet?’*  *‘need to think how to present the evidence to senior management that this is a good idea’* | (Research) Explore the effectiveness of MECC on the outcomes of service users using both quantitative and qualitative methods | Randomised control trial comparing MECC conversations with usual care control, with a process evaluation |
