# Supplementary material for: Mapping regional implementation of ‘Making Every Contact Count’: mixed-methods evaluation of implementation stage, strategies, barriers and facilitators of implementation
Source: BMJ Open. 2024 Jul 22;14(7):e084208. doi: 10.1136/bmjopen-2024-084208 (PMC11268057; doi:10.1136/bmjopen-2024-084208)
Supplement: online supplemental file 7 [file bmjopen-14-7-s007.pdf]

Supplementary Material 7: APEASE scores for each initial recommendation, prior to discussion and amendments.

Recommendation	A	P	E	A	S	E	Total
**Recommendation 1**: Create a support mechanism to put in place to support and maintain training cascading after receiving MECC training – potentially build support into regional offer.	15	16	16	14	11	8	80
**Recommendation 2**: Create a standardised way to record/monitor MECC implementation/delivery across the region (to provide more structure) - how to embed within current systems – ensure everyone capturing the same data to improve measurability	14	7	12	12	11	9	65
**Recommendation 3**: Implement further staff resources to combat staff capacity/time to deliver/implement issues	10	10	10	5	10	10	55
**Recommendation 4**: Managing competing priorities: Implement organisational lead for MECC within organisation with sole responsibility to drive MECC implementation/delivery forward (admin support)	12	11	11	6	11	9	60
**Recommendation 5**: Incorporate the use/further of PPI to ensure clients/service users are being delivered MECC as intended – collect qual feedback to provide evidence of implementation effectiveness (i.e., case studies)	14	12	13	10	9	8	66
**Recommendation 6**: Encourage further ‘buy in’ of MECC from additional pathways/organisational departments by disseminating evidence of effectiveness of the programme.	16	14	15	13	11	11	80
**Recommendation 7**: Further develop/utilise online training/networking model to increase uptake in MECC training and networking opportunities (reduce inequalities i.e., travel requirements)	15	13	17	18	12	10	85
**Recommendation 8:** Ensure core MECC resources are easily accessible and provide short e-learning on how to use these resources and how to tailor for organisational fit without losing the consistency of the MECC message.	17	14	13	14	11	11	80
**Recommendation 9**: Implement MECC throughout organisational policies to ensure good practice and raise awareness of the MECC programme (i.e., staff inductions, return to work interviews)	12	8	15	13	11	12	71
