# Supplementary material for: Mapping regional implementation of ‘Making Every Contact Count’: mixed-methods evaluation of implementation stage, strategies, barriers and facilitators of implementation
Source: BMJ Open. 2024 Jul 22;14(7):e084208. doi: 10.1136/bmjopen-2024-084208 (PMC11268057; doi:10.1136/bmjopen-2024-084208)
Supplement: online supplemental file 9 [file bmjopen-14-7-s009.pdf]

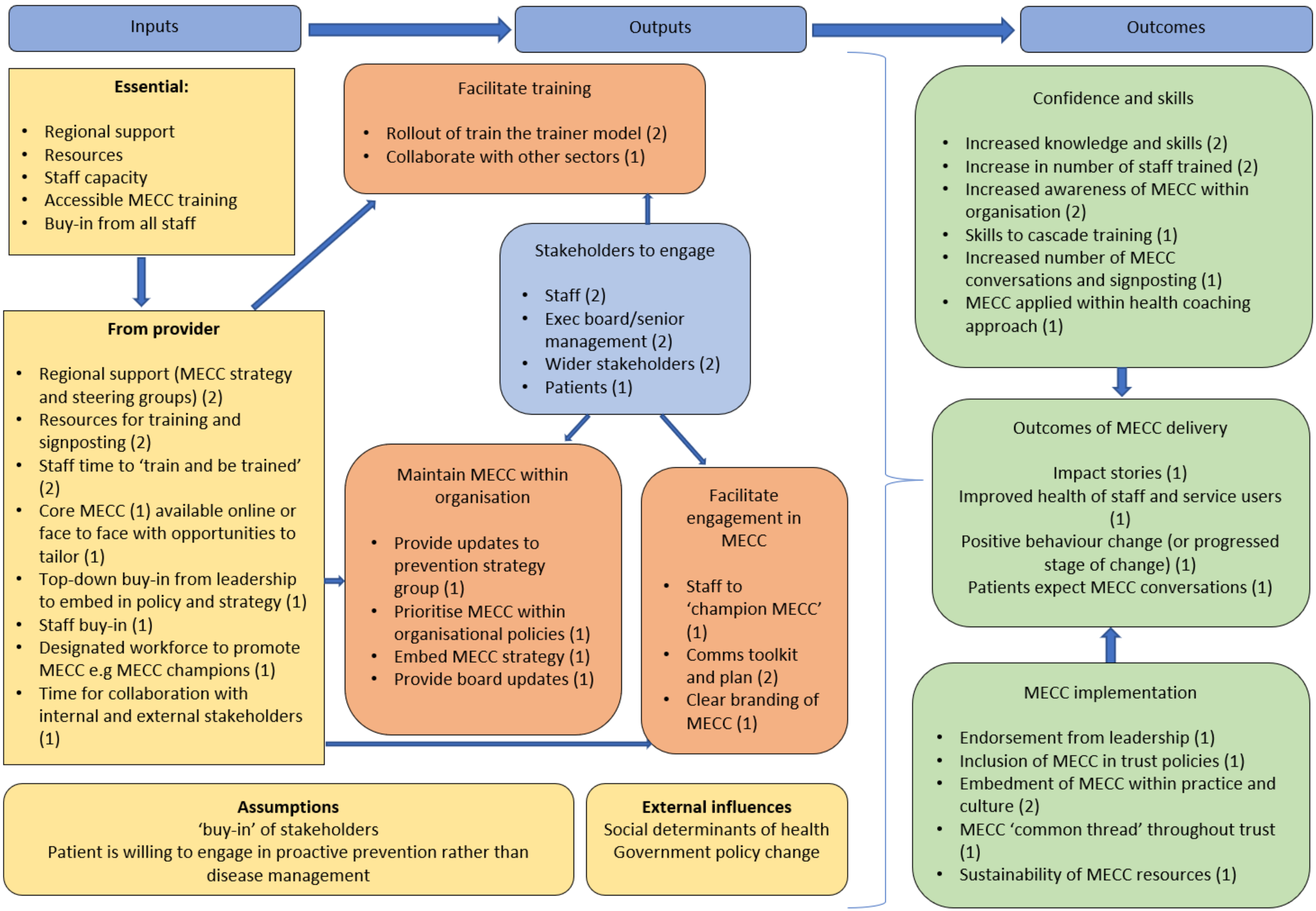

Figure 2: Template logic model for healthcare, as a composite of logic models created during workshop 3. Elements are presented in order of frequency, with most commonly cited elements at the top of each section. Frequencies of occurrences across each logic model (N = 2) of elements are displayed in brackets.
