# Supplementary material for: Mapping regional implementation of ‘Making Every Contact Count’: mixed-methods evaluation of implementation stage, strategies, barriers and facilitators of implementation
Source: BMJ Open. 2024 Jul 22;14(7):e084208. doi: 10.1136/bmjopen-2024-084208 (PMC11268057; doi:10.1136/bmjopen-2024-084208)
Supplement: Supplementary file 10 [file bmjopen-2024-084208.reviewer_comments.pdf]

PEER REVIEW HISTORY

BMJ Open publishes all reviews undertaken for accepted manuscripts. Reviewers are asked to complete a checklist review form (**http://bmjopen.bmj.com/site/about/resources/checklist.pdf**) and are provided with free text boxes to elaborate on their assessment. These free text comments are reproduced below.

ARTICLE DETAILS

**TITLE (PROVISIONAL)**	Mapping regional implementation of 'Making Every Contact Count': mixed methods evaluation of implementation stage, strategies, barriers and facilitators of implementation
**AUTHORS**	Rodrigues, Angela; Nichol, Bethany; Wilson, Rob; Charlton, Caroline; Gibson, Beckie; Harrison, Deborah; Orange, Denise; Robson, Craig; Finch, Tracy; Haighton, Catherine; Maniatopoulos, Gregory; Giles, Emma; Harland, Jill

VERSION 1 – REVIEW

**REVIEWER**	Elissa Faro<br>The University of Iowa Roy J and Lucille A Carver College of Medicine, Internal Medicine
**REVIEW RETURNED**	19-Feb-2024

**GENERAL COMMENTS**	Thank you for the opportunity to review this manuscript, which presents interesting research on the implementation of an intervention at scale across one region. In general, the theoretical framing is helpful, but the lack of detail around data collection and analysis makes it very hard to follow the thread and understand what produced the findings and place them in context. Specific comments by section are listed below.<br><br>Introduction<br>• Page 4 line 11 – clinicians are delivering behavioral change interventions with patients, presumably, not behavioral change with patients<br>• Page 5 paragraph starting at line 11 – 8 different studies doesn't feel like a paucity of research into the implementation; perhaps qualify that a bit more? Like there has been limited research focused on implementer perceptions or something like that; or do you mean theoretically-informed research?<br>• Page 6 paragraph starting line 3 – same as above – you've just described several studies that report on what support is need to foster implementation process; I would start paragraph with second sentence<br><br>Methods<br>• Page 8 line 3 – where is public involvement in the study design diagram?<br>• Page 9 line 6 – how does the NENC MECC Strategy Group differ from the NENC MECC Interest Group mentioned in previous paragraph?<br>• Page 10 line 8 – what is the "high-level implementation data" mentioned here?<br>• Page 11 – the way the group discussions are presented is confusing; why are numbers included for the third group but not the

	first two? Why did the first two have written answers but not the third? Were the first two virtual? The methods need to be described in more detail<br>• Page 11 – Please include detail about the interviews – recruitment, sampling, data collection methods, etc.<br>• Page 12 line 21 – is the headline findings meeting with a group of interviewees head in October 2022 the same as the third group discussion held in October 2022 mentioned above? If not, please clarify the difference in participation<br>• Page 13 line 7-8 – who participated in the stakeholder workshop? Were they different or same to group discussions, surveys, and/or interviews?<br><br>Findings<br>• Generally, it would be stronger if some of the results were shared more granularly – what are the results of the “Exploratory descriptive and inferential analyses”? Quotations from the qualitative data would be helpful as well<br>• Enablers and Barriers to MECC implementation – perhaps break this section into subheadings; it’s too hard to follow as written<br>• Page 17 – Please make clear in your presentation of findings when you switch from presenting findings from just document analysis and survey (page 13 through 16) to group discussions and interviews starting on page 17<br>• Optimisation and sustainability of MECC implementation – need to change the heading level so that it’s clear that these are the results from a different set of methods; it’s hard to refer back to the methods for the process of collating the logic models – may help to remind the reader from which organizations, who was involved in this analysis, etc.<br><br>Discussion<br>• Strengths and limitations – You may want to clarify earlier in the paper how, in what numbers, in which data collection, analysis, and dissemination activities the MECC regional strategy group participated, perhaps with a figure

**REVIEWER**	Oonagh Meade<br>National University of Ireland Galway
**REVIEW RETURNED**	11-Mar-2024

**GENERAL COMMENTS**	This was a comprehensive multi-method study exploring the regional implementation of Making Every Contact Count in England. The introduction clearly sets out the need for the study within the context of relevant literature and the study aim is clearly described.<br><br>The methods are described well overall. A couple of clarifications would be helpful on the following items:<br><br>-It would be good to explain a little more what was meant by this sentence - "This part of the study was also informed by high-level implementation data collected by the MECC coordinator (CR) (retrieved from the local 9 integrated care system training app) and collated in an anonymous overview document shared with the 10 research team". Could the authors say a little more about how this overview document supported the documentary analysis?<br><br>-It would be good to mention what types of stakeholders were part of the MECC strategy group mailing list in this section of the methods - "All stakeholders who signed up for the MECC strategy group

	mailing list (n = 86 stakeholders) were invited to take part in the online survey via e-mail invitations from the MECC Strategy group between 15 June 2022 to September 2022." Pg9<br><br>-It would be good to describe what a padlet is for those who might not be familiar (pg10).<br><br>-It would be good to clarify if the participants were different for the group discussions and individual interviews - i.e. did participants chose between each mode of participation?<br><br>-It would also be helpful to clarify in the methods if the aim of the interviews and group discussion were the same and if the same topic guide was used?<br><br>-It would be useful to clarify if the thematic framework was adjusted at all after the analysis of the first three interviews - "First, a map of all the themes that emerged from the interviews (from an initial sample of three transcripts) was created. This initial framework was then applied to all transcripts until no new codes were retrieved from the final transcripts (43)".<br><br>-When reading the sentence below I was unsure about whether the analysis of the interview data was done deductively according to the TDF and NOMAD domains or inductively? "To identify key TDF and NPT domains influencing the implementation of MECC, themes were prioritised in relation to the number of transcripts that contained the domains (as an indicator of relevance), the number of themes generated within each domain (as an indicator of elaboration), triangulation between sub-themes across interviews and group discussions within each domain and level of agreement with NPT domains derived from NoMAD survey items (as corroborators of findings), and conflict within domains (as an indicator of coherence)". It would be good to clarify this process.<br><br>Overall the results were clearly presented with sufficient elaboration in the supplementary files. Two suggestions are made below:<br><br>-For the survey data - is it possible to say how many organisations the 34 staff represented?<br><br>-It would also be good to clarify how many of the participants were working in patient-facing roles if possible as this might explain the lower response to the survey items on MECC delivery.<br><br>-In supplementary file 4 it would be good to list the COM-B items fully as you have done for the NOMAD items just to help in interpretation.<br><br>The discussion was clearly written with good consideration of the strengths and weaknesses of the work and clear links to existing evidence on the implementation of brief interventions.

VERSION 1 – AUTHOR RESPONSE

**REVIEWER 1**		
Thank you for the opportunity to review this manuscript, which	Thank you for the helpful comments provided. We have done our utmost to articulate how this	Throughout the

presents interesting research on the implementation of an intervention at scale across one region. In general, the theoretical framing is helpful, but the lack of detail around data collection and analysis makes it very hard to follow the thread and understand what produced the findings and place them in context. Specific comments by section are listed below.	study was conducted and how the study's results contribute not only to the literature but more importantly to policy and practice. These revisions can be found throughout the manuscript highlighted in red.	document (highlighted in red)
Introduction<br>• Page 4 line 11 – clinicians are delivering behavioral change interventions with patients, presumably, not behavioral change with patients<br><br>• Page 5 paragraph starting at line 11 – 8 different studies doesn't feel like a paucity of research into the implementation; perhaps qualify that a bit more? Like there has been limited research focused on implementer perceptions or something like that; or do you mean theoretically-informed research?<br><br>• Page 6 paragraph starting line 3 – same as above – you've just described several studies that report on what support is need to foster implementation process; I would start paragraph with second sentence	Thank you, we have revised the sentence accordingly.<br><br>Thank you for your feedback. We appreciate your suggestion to provide further qualification regarding the research gap. We revised the manuscript to clarify that there is a lack of evidence on effectiveness and that the use of various models of MECC gives us contrasting perspectives of implementation success. We have revised the text to: "MECC lacks a universal approach, as organisations adopt diverse strategies, potentially contributing to a scarcity of evidence regarding its effectiveness and to disparities in implementation success."<br><br>Thank you for your suggestion, we have revised accordingly.	Page 3, line 11<br><br>Page 4, line 11-12<br><br>Page 5, line 4.
Methods<br>• Page 8 line 3 – where is public	Thank you for this valid comment. The lay	Page 7 line

involvement in the study design diagram?	advisory groups and MECC strategy group have been added to Figure 1.	1-3
• Page 9 line 6 – how does the NENC MECC Strategy Group differ from the NENC MECC Interest Group mentioned in previous paragraph?	Thanks for spotting the inconsistency, we have revised all references to NENC MECC Strategy Group.	Page 7, line 7-11<br><br>Page 9, line 23 and Page 10, line 1-2
• Page 10 line 8 – what is the "high-level implementation data" mentioned here?	Thank you, we have added a brief description of what this entails. More precisely, we added the following: "This high-level implementation data included details on the following: stage of implementation, liaison meeting, existence of a logic model and/or implementation plan, and training delivery (number of attendees/date of delivery)."	Page 11, line 3-11
• Page 11 – the way the group discussions are presented is confusing; why are numbers included for the third group but not the first two? Why did the first two have written answers but not the third? Were the first two virtual? The methods need to be described in more detail	Thank you, we have clarified within the manuscript why we didn't present the participant settings numbers due to not collecting this data since group discussions 1 & 2 represented a small number of participants. We have also clarified that the first two group discussions were virtual therefore using the padlet as an aid for facilitation.	Page 11, line 18-23
• Page 11 – Please include detail about the interviews – recruitment, sampling, data collection methods, etc.	Thank you, we have added in further details surrounding the interviews. More precisely, we added the following: "As above, members of the MECC strategy group mailing list were invited to take part in one-to-one interviews via email invitations. Recruitment was targeted to collect a purposive sample, again within setting and the stage of implementation. Several participants had taken part in a group discussion previously before interview (although not all). Interviews were conducted by (CC = 17) and (AR =1)	Page 13, line 3-4

• Page 12 line 21 – is the headline findings meeting with a group of interviewees head in October 2022 the same as the third group discussion held in October 2022 mentioned above? If not, please clarify the difference in participation<br><br>• Page 13 line 7-8 – who participated in the stakeholder workshop? Were they different or same to group discussions, surveys, and/or interviews?	online via Microsoft Teams, with interviews ranging between 35 minutes to 1 hour 30 minutes in length."<br><br>Thank you for pointing this out. The headline findings meeting, and the third group discussion took place on the same day, in distinct sessions. We have amended the manuscript to reflect this with the following: "Within the third group discussion (held October 2022), headline findings were presented to a group of interviewees to check whether the data interpretation matched their understanding of MECC implementation, providing an opportunity for further data confirmation through discussion."<br><br>Thank you. As above the participants were the same as those within the third group discussion due to them being the same event. We have amended the text to reflect this: "To enhance the suitability and acceptability of these recommendations, the third group discussion was conducted by collecting data from 19 stakeholders (interviewees) (total of 85min)."	Page 13, line 13-14
<u>Findings</u><br>• Generally, it would be stronger if some of the results were shared more granularly – what are the results of the "Exploratory descriptive and inferential	Thank you for this suggestion. The themes presented were developed from integrating all sources of data to provide a comprehensive overview of MECC implementation across the NENC. Subsequently, after consideration, we	

analyses"? Quotations from the qualitative data would be helpful as well<br><br>• Enablers and Barriers to MECC implementation – perhaps break this section into subheadings; it's too hard to follow as written.	do not think it would be meaningful to separate the findings by source of data, particularly due to the low response rate for the survey and document analysis. Within the results, the write-up has been revised to clearly indicated data sources (e.g survey) throughout. We have amended the methods section for the survey data to more clearly explain this process. We have also labelled each heading with the source/s of data that informed it. Quotations relevant to barriers and facilitators are presented in Table 3 due to the existing complexity of the paper. Given that the findings section is already extensive, preserving the quotations within the Table streamlines this section. However, we have also added a quotation to frame the section 'Optimisation and sustainability of MECC implementation' give that it was also informed by qualitative data.	Page 10, line 17-20<br><br>Page 22, line 7-11<br><br>Page 14-27<br><br>Page 14-27
• Page 17 – Please make clear in your presentation of findings when you switch from presenting findings from just document analysis and survey (page 13 through 16) to group discussions and interviews starting on page 17	Thank you for this helpful suggestion. This section has now been split into three subheadings; 1) key domains of influence, 2) enablers to MECC implementation, and 3) barriers to MECC implementation.<br><br>Thank you, we have improved the consistency of reporting by clearly stating the data sources for the specific findings being highlighted.	Page 14, line 2; Page 22, line 3
• Optimisation and sustainability of MECC implementation – need to change the heading level so that it's clear that these are the results from a different set of methods; it's hard to refer back to the methods for the process of collating the logic models – may help to remind the reader from which organizations, who was involved in this analysis, etc.	Thank you for this valid comment. We have amended the subheadings of the methods and results to separate the 'triangulation of mixed methods' from the 'development of recommendations to support future	

	implementation'. We feel this has helped to clarify which methods informed which set of findings.	
Discussion<br>• Strengths and limitations – You may want to clarify earlier in the paper how, in what numbers, in which data collection, analysis, and dissemination activities the MECC regional strategy group participated, perhaps with a figure	Thank you for this valid comment. We have added the input of the MECC strategy group to Figure 1 along with the input of the Lay Advisory Groups (LAGs).	Page 7 line 1-3
**REVIEWER 2**		
This was a comprehensive multi-method study exploring the regional implementation of Making Every Contact Count in England. The introduction clearly sets out the need for the study within the context of relevant literature and the study aim is clearly described.	Thank you so much for the overall positive feedback and for your encouraging comments.	--
The <u>methods</u> are described well overall. A couple of clarifications would be helpful on the following items:<br>-It would be good to explain a little more what was meant by this sentence - "This part of the study was also informed by high-level implementation data collected by the MECC coordinator (CR) (retrieved from the local 9 integrated care system training app) and collated in an anonymous overview document shared with the 10 research team". Could the authors	Thank you, we have added a brief description of what this entails. More precisely, we added the following: "This high-level implementation data included details on the following: stage of implementation, liaison meeting, existence of a logic model and/or implementation plan, and training delivery (number of attendees/date of delivery)."	Page 9, line 23 and Page 10, line 1-2

say a little more about how this overview document supported the documentary analysis?		
-It would be good to mention what types of stakeholders were part of the MECC strategy group mailing list in this section of the methods - "All stakeholders who signed up for the MECC strategy group mailing list (n = 86 stakeholders) were invited to take part in the online survey via e-mail invitations from the MECC Strategy group between 15 June 2022 to September 2022." Pg9	Thank you, to improve clarity we have added the following text: "The mailing list includes representatives from the following sectors: local authorities/government, NHS healthcare trusts, emergency services, NHS integrated care board managers, and academics."	Page 10. Line 7-9
-It would be good to describe what a padlet is for those who might not be familiar (pg10).	Thank you, we have added a brief definition of padlet and the following text was added to the manuscript: "During the first and second group discussions (held virtually, via Microsoft Teams), Padlet, an online interactive platform designed to simulate a virtual notice board, was used to facilitate discussion and document written responses."	Page 11, line 1-3
-It would be good to clarify if the participants were different for the group discussions and individual interviews - i.e. did participants chose between each mode of participation?	Thank you, we have added in a brief sentence to clarify which participants took part in which parts of the project. More specifically, we added the following text to the manuscript: "As all members of the MECC strategy mailing list were invited to take part in both the group discussions and the interviews, five participants took part in both aspects of the study."	Page 11, line 15-19
-It would also be helpful to clarify in the methods if the aim of the interviews and group discussion were the same and if the same topic guide was used?	Thank you, we have clarified the aims of both the group discussions and the interviews, and whether the same topic guide was utilised for both with the following added to the manuscript "The interactive platform provided a series of questions based upon the group discussion topic guide (see additional file 1) which aimed to prompt responses around planning and	Page 11, line 3-6; Page 11, line 21

	implementation, service reach, relationships with other organisations, and interventions delivered."<br>and<br>"Topics addressed within the interview topic guide aimed to elicit responses on the participant's role in MECC implementation, what MECC means to them, recalling early implementation, motivations for implementation, acceptability of MECC, support and resources, the influence of other organisations, current stage of implementation, impact and outcomes of MECC, and how implementation could be improved."	
-It would be useful to clarify if the thematic framework was adjusted at all after the analysis of the first three interviews - "First, a map of all the themes that emerged from the interviews (from an initial sample of three transcripts) was created. This initial framework was then applied to all transcripts until no new codes were retrieved from the final transcripts (43)".	Thank you for your comment. We have provided further clarification on our data analysis process to fully describe how we used the framework approach in this research. We added the following text: "We employed an inductive thematic analysis method, allowing themes to emerge from the data, a common practice in TDF studies (45). To analyse the interview transcripts and identify themes, we used the framework method (46). The transcripts underwent multiple readings by researchers (CC, BN, BG, AR) and were meticulously coded line by line to discern patterns. After an initial inductive coding of a subset transcripts (n=3), a coding framework was created and mapped onto NPT and TDF domains. Subsequent analysis of all transcripts was deductive according to the created coding framework, with adjustments made as necessary. "	Page 12, line 14-20
When reading the sentence below I was unsure about whether the analysis of the interview data was done deductively according to the TDF and NOMAD domains or inductively? "To identify key TDF and NPT domains influencing the	Thank you for your comment. We have provided further clarification on this aspect. We added the following text: "We added the following text: "We employed an inductive thematic analysis method, allowing themes to emerge from the data, a common practice in TDF studies (45). To analyse the interview transcripts and identify	Page 12, line 14-20

implementation of MECC, themes were prioritised in relation to the number of transcripts that contained the domains (as an indicator of relevance), the number of themes generated within each domain (as an indicator of elaboration), triangulation between sub-themes across interviews and group discussions within each domain and level of agreement with NPT domains derived from NoMAD survey items (as corroborators of findings), and conflict within domains (as an indicator of coherence)". It would be good to clarify this process.	themes, we used the framework method (46). The transcripts underwent multiple readings by researchers (CC, BN, BG, AR) and were meticulously coded line by line to discern patterns. After an initial inductive coding of a subset transcripts (n=3), a coding framework was created and mapped onto NPT and TDF domains. Subsequent analysis of all transcripts was deductive according to the created coding framework, with adjustments made as necessary."	
Overall the <u>results</u> were clearly presented with sufficient elaboration in the supplementary files. Two suggestions are made below:<br>-For the survey data - is it possible to say how many organisations the 34 staff represented?	Thank you for this suggestion. To retain anonymity of survey participants, the specific organisation was not collected, only their sector. Therefore, we have acknowledged this as a limitation in the 'strengths and limitations' section of the manuscript.	Page 33, line 1-7
-It would also be good to clarify how many of the participants were working in patient-facing roles if possible as this might explain the lower response to the survey items on MECC delivery.	Thank you for this interesting observation. We have added the following to the findings to reflect this; '*Only three participants responded that their organisation was involved in MECC delivery indicating a lack of representation of patient-facing roles, perhaps explaining the low response rate for survey items related to MECC delivery.' We have also acknowledged this limitation in the discussion section.*	Page 14, line 14-16<br><br>Page 33, line 1-7
-In supplementary file 4 it would be good to list the COM-B items fully as you have done for the NOMAD items just to help in interpretation.	Thank you, we have added those details as requested.	Supplement file 4
The discussion was clearly written with good consideration of the strengths and weaknesses of the	Thank you for your kind feedback on the discussion section.	--

work and clear links to existing evidence on the implementation of brief interventions.		

VERSION 2 – REVIEW

**REVIEWER**	Elissa Faro<br>The University of Iowa Roy J and Lucille A Carver College of Medicine, Internal Medicine
**REVIEW RETURNED**	03-May-2024

**GENERAL COMMENTS**	Thank you for the opportunity to review this manuscript. In general, systemic evaluations of a large-scale complex implementation are interesting to the audience and would be helpful for others undertaking similar work, which is more and more common. However, the lack of clarity and detail in the current draft make it challenging to understand.<br><br>The primary issue with the current manuscript is the opacity of the data collection and analysis methods. It's unclear the order in which the data collection activities occurred and then how the ongoing analysis informed each activity - perhaps a timeline figure might clarify this for the reader. Another figure that might be helpful is a framework or model for how the activities connect to different conceptual elements. Across both the methods and results sections, it's unclear how many organizations are represented, which makes the generalizability of the results difficult to determine. For example, the results are reported by data source, as in "two documents reported x implementation barrier" which makes it further difficult to determine. Additionally, some of the analyses and results are not clearly described - for example, the results of the third discussion group rating exercise.<br><br>The results section might be strengthened with additional quotations, given the majority of the data is qualitative. The tables are comprehensive, but additional detail in the narrative text itself might be helpful given the amount of information included in the tables.<br><br>The discussion section could situate the findings within the current implementation literature more clearly, in order to help the reader understand how these results and this instance of implementation differ or are similar to other projects.

VERSION 2 – AUTHOR RESPONSE

**Reviewer Comment**	**Response**	**Page no/line no**
**REVIEWER 1**		
Reviewer: 1<br>Thank you for the opportunity to review this manuscript. In general, systemic evaluations of a large-	Thank you for your valuable feedback. We appreciate the time and effort you have taken to review our manuscript. We have carefully considered your comments and made revisions	Throughout the manuscript

scale complex implementation are interesting to the audience and would be helpful for others undertaking similar work, which is more and more common. However, the lack of clarity and detail in the current draft make it challenging to understand.	based on your suggestions.	
The primary issue with the current manuscript is the opacity of the data collection and analysis methods. It's unclear the order in which the data collection activities occurred and then how the ongoing analysis informed each activity - perhaps a timeline figure might clarify this for the reader. Another figure that might be helpful is a framework or model for how the activities connect to different conceptual elements.	We agree with the Reviewers that more clarity on the data collection and analysis methods is needed. We have added another figure noting the timeline of events and the purpose of each activity (see Figure 2). We have also added the guiding frameworks for each work packages to Figure 1.	Page 6-7, & attached Figures 1& 2
Across both the methods and results sections, it's unclear how many organizations are represented, which makes the generalizability of the results difficult to determine. For example, the results are reported by data source, as in "two documents reported x implementation barrier" which makes it further difficult to determine.<br><br>Additionally, some of the analyses and results are not clearly described - for example, the results of the third discussion group rating exercise.	Thank you for your comment. We have added in information to the methods section disclosing how many organisations were involved within the interview stage (specific organisation data was not collected during workshops 1 & 2/online survey). We have also within the results further clarified the documentary analysis results by providing how many organisations they relate to and not just the number of documents. We hope this provides further clarity.<br><br>Thank you for the comment around providing further clarification about the third group discussion. More information about the procedure of the group discussion has been added to the methods section, and more information on how the findings were analysed and triangulated has been added to the results section.	Page 10-11 (methods section); Page 14-16 (findings section)<br><br>Page 10, line 17-24; Page 11, line 1-3; Page 13, line 6-7
The results section might be	Thank you for this suggestion, we have added	Page 14-

strengthened with additional quotations, given the majority of the data is qualitative. The tables are comprehensive, but additional detail in the narrative text itself might be helpful given the amount of information included in the tables.	quotes throughout the results section where the source data was qualitative. Some of the added quotes have been taken from Tables 3 and 4, so we have re-submitted these revised tables too.	23
The discussion section could situate the findings within the current implementation literature more clearly, in order to help the reader understand how these results and this instance of implementation differ or are similar to other projects.	Thank you for this helpful comment. We have done our utmost to situate our findings within relevant implementation literature more clearly throughout the discussion section of this manuscript.	Page 23-30

VERSION 3 – REVIEW

**REVIEWER**	Elissa Faro<br>The University of Iowa Roy J and Lucille A Carver College of Medicine, Internal Medicine
**REVIEW RETURNED**	26-Jun-2024

**GENERAL COMMENTS**	The revisions have significantly improved the clarity and understandability of the manuscript.
